# Study of the survival of patients with head and neck cancer in relation to Circulating Tumor Cells (CTCs)

**DOI:** 10.1371/journal.pone.0320485

**Published:** 2025-04-01

**Authors:** Romina Mastronicola, Elise Kayser, Pauline Le Roux, Agathe Barrat, Alexandre Aubertin, Aurore Casse, Léa Nominé, Hélèna Villard, Sophie Cortese, Emilie Beulque, Jean-Louis Merlin, Gilles Dolivet

**Affiliations:** 1 Institut de Cancérologie de Lorraine ICL, Vandœuvre-lès-Nancy, France; 2 CRAN, CNRS, UMR, Université de Lorraine, Vandœuvre-lès-Nancy, France; The Ohio State University, UNITED STATES OF AMERICA

## Abstract

Distant metastasis in head and neck cancer are one of the first factors contributing to death. Currently, it is difficult to detect them early with our conventional techniques such as Positron Emission Tomography scanner (PET-scanner) and Magnetic Resonance Imaging (MRI). Therefore, it is important to find new markers that can help us in the care of the patient. This study aimed at comparing two methods (Reverse Transcription-Polymerase Chain Reaction and CellSearch) to detect circulating tumor cells (CTC) as a prognosticator. Results were statistically significant for markers EphB4 (p-value =  0.0003), CEA (p-value =  0.0006), CK 18 (p-value =  0.0011) and Ep-CAM (p-value =  0.0299) and demonstrate that our detection techniques could be used by optimizing our protocol. In addition, results of the rate of CTCs helped identify this as an indicator of a prognosis for the patient. Indeed, the study revealed that most patients in remission exhibited a decrease in post-operative CTCs, whereas patients experiencing relapses demonstrated an increase in CTCs, which was correlated with a poor prognosis.

## 1. Introduction

Head and Neck Cancer is the 6^th^ most common cancer in the world and the 8^th^ leading cause of mortality [1]. Patient prognosis is influenced by factors such as age, human papillomavirus status and smoking or alcohol consumption [2,3]. This type of cancer can cause distant metastasis in the lung, bone and liver mostly, but the incidence is considered low with 3 to 50% of clinically identified distant localizations [4]. However, chance of survival for patient with distant metastasis are low as metastasis in cancer patients is a leading cause of death [5]. Nowadays, the imaging techniques currently used like Positron Tomography Emission-scanner (PET-scanner) and Magnetic Resonance Imaging (MRI) do not detect early relapses [6]. Early relapses refer to relapses appearing within one year after the treatment of the primitive lesion. It is therefore crucial to use new markers that can predict patient survival and improve the prediction of locoregional recurrence and distant metastasis in squamous cell carcinoma of head and neck (SCCHN) patients. The metastatic process is closely related to the colonization of distant organs by circulating tumor cells (CTCs). They can be found spontaneously in the blood or following surgery. Indeed, manipulation of the tumor during surgery can lead to detachment of tumor cells from their primary tumor site [7]. This is made possible by an epithelial-mesenchymal transition (EMT), giving epithelial cells mesenchymal-like properties [8]. This transition generates cells endowed with cellular mobility and invasion capacities, thus facilitating dissemination [9]. Tumor cells can then reach blood or lymphatic circulation. By invading the lymphatic system, these cells will be led into the lymph nodes and settle there. Therefore, tumor cells circulating in the bloodstream will be able to disseminate in different organs [10]. The dissemination of CTCs can be responsible for post-operative micrometastasis, which can increase the risk of metastasis development.

In head and neck cancer, the major limitation to detect early distant metastasis is the lack of routine screening method. An increasing number of studies have shown interest in liquid biopsy analyzing CTCs to detect and characterize minimal residual disease (MRD). MRD reflects both the presence of tumor cells disseminated to organs distant from the primary tumor, or the persistence of residual tumor cells after surgery [11–14]. Also, previous studies have shown a correlation between recurrence and/or metastasis rates and CTC positivity in patients with SCCHN [15,16]. The presence of CTCs therefore indicates a poor prognosis and could impact patient survival [17–19].

Such studies were conducted on breast cancer, prostate, colon, even lung cancer yet such a few were conducted on head and neck cancer. Since 90% of head and neck cancers are of squamous cell type [20], we interested ourselves especially on 9 molecules expressed in this type of cancers: BphB4, Elf3, CEA, CK18, CK19, Ep-CAM, EGFR, PVA, SCCA, GAPDAH (reference gene). The aim of our study was to detect the presence of CTCs in head and neck epidermoid cancers and to evaluate their variation rate in blood circulation before, during and after a surgical act. The second aim of our study was to compare the results obtained by two different techniques: Reverse Transcription-Polymerase Chain Reaction (RT-PCR) and CellSearch. CellSearch is an automaton which has been approved by the Food and Drug Administration (FDA) to detect circulating tumor cells in metastatic breast cancer [21]. The detection and analysis of CTCs in such diseases allows the medical team to give an early diagnosis and to adapt the patient’s treatment [22].

We evaluated the use of CTCs detection during the surgical procedure as a prognostic factor of local, regional and/or metastatic relapse of head and neck cancer in stages III and IV. This protocol will allow us to validate the research of CTCs in clinical situations and then to develop prospective and diagnostic studies on CTCs.

## 2. Materials and methods

### 2.1. Patients characteristics

Patients were recruited for clinical research (EudraCT N°: 2010-A00586-33, approved by the regional ethics committee “Comité de Protection des Personnes Est III”). Between April 2014 and April 2015, we conducted a study at the Institute of Cancerology of Lorraine (ICL) focusing on the detection of CTCs after surgery in patients with SCCHN stage III or IV. The staging edition used was the Tumor Node Metastasis (TNM) classification. Forty patients were included in our study. All patients were diagnosed with SCCHN and underwent surgery at the ICL. They received a radiological diagnosis and had never undergone chemotherapy or radiotherapy treatment beforehand. Informed consent was obtained from all subjects involved in the study.

Every patient presented with a squamous cell carcinoma: 26 of them had a well-differentiated carcinoma, 9 had a moderately differentiated one, and 5 had a poorly differentiated one. 3 patients presented no trace of smoking, and 4 of them presented traces of smoking yet had stopped for 15 years. 14 patients presented no alcoholic intoxication. Regarding the localization of the tumors, 10 patients had larynx cancer, 12 of them in the oropharynx area, 11 of them in the buccal cavity and 7 of them in the pharyngo-larynx area. 19 patients had metastatic adenopathy with capsular rupture, 12 had metastatic adenopathy without capsular rupture, and 9 had no lymph node metastasis. All patients had a M0 status. Datas are summed up in Table 1. Every patient got a classical pre-therapeutical tumoral examination: a cervical-thoracic scanner with the injection of a contrast product, a fluorodeoxyglucose-PET-scanner +/- MRI. Everything was done approximately 30 days before the surgical care, as well as a panendoscopy approximately 15 days before. Blood samples were drawn from all patients one day before surgery (D-1), two hours after surgery (D+0) and seven days after surgery (D+7). All these samples were used to detect CTCs using two methods: real-time quantitative PCR and the CellSearch system.

**Table 1:** Clinical characteristics of patients; W and M refer to woman and man. In the differentiation column, 1 refers to poorly differentiated cancers, 2 to moderately differentiated cancers, and 3 to well-differentiated cancers. As said above, adenopathy could have been with or without capsular rupture.

Patients	Sex	Type of cancer	Differentiation	Type of adenopathy	Surgical margins (mm)	T stage	Lymph node stage
1	W	Larynx	1	–	4	III	N0
2	M	Oropharynx	2	No caps. rupture	4	III	N1
3	M	Pharyngo-larynx	3	Caps. rupture	5	IV	N2
4	M	Larynx	2	No caps. rupture	3	III	N1
5	M	Pharyngo-larynx	3	No caps. rupture	3	IV	N3
6	M	Oropharynx	2	–	3	IV	N0
7	W	Buccal cavity	3	Caps. rupture	4	III	N1
8	W	Buccal cavity	3	Caps. rupture	5	III	N2
9	M	Oropharynx	3	No caps. rupture	4	III	N1
10	M	Larynx	2	–	5	III	N0
11	M	Larynx	3	Caps. rupture	3	IV	N3
12	M	Oropharynx	2	No caps. rupture	4	IV	N1
13	M	Oropharynx	3	No caps. rupture	3	IV	N3
14	M	Buccal cavity	3	Caps. rupture	4	III	N2
15	M	Pharyngo-larynx	2	No caps. rupture	5	III	N3
16	M	Oropharynx	3	Caps. rupture	4	III	N2
17	M	Larynx	3	–	3	III	N0
18	W	Larynx	2	Caps. rupture	4	III	N1
19	W	Buccal cavity	2	Caps. rupture	3	III	N2
20	W	Larynx	3	No caps. rupture	4	III	N3
21	M	Oropharynx	3	Caps. rupture	3	III	N2
22	M	Oropharynx	1	No caps. rupture	4	IV	N1
23	M	Pharyngo-larynx	3	Caps. rupture	5	IV	N2
24	M	Larynx	3	Caps. rupture	4	III	N3
25	M	Larynx	3	–	3	III	N0
26	M	Larynx	3	–	4	III	N0
27	M	Oropharynx	3	Caps. rupture	5	IV	N1
28	W	Buccal cavity	3	No caps. rupture	4	IV	N2
29	M	Buccal cavity	1	Caps. rupture	3	III	N2
30	M	Pharyngo-larynx	1	No caps. rupture	4	III	N3
31	M	Pharyngo-larynx	2	Caps. rupture	5	III	N1
32	M	Buccal cavity	3	No caps. rupture	3	III	N2
33	M	Pharyngo-larynx	3	–	3	III	N0
34	M	Oropharynx	3	Caps. rupture	4	IV	N1
35	M	Buccal cavity	1	–	3	IV	N0
36	M	Oropharynx	3	Caps. rupture	4	III	N1
37	M	Buccal cavity	3	Caps. rupture	3	IV	N2
38	M	Buccal cavity	3	Caps. rupture	3	III	N1
39	M	Buccal cavity	3	Caps. rupture	4	IV	N3
40	M	Oropharynx	3	–	4	IV	N0

### 2.2. Cells detection

CTCs were detected by two different methods: the CellSearch system and real-time quantitative PCR. Cellsearch is one of the immunological methods allowing the detection of CTCs in peripheral blood. To detect these cells, we used the CellSearch epithelial kit and EGFR. It is a semi-automated and highly reproducible method relying on the fact that epithelial cells are normally absent in blood. Thus, any epithelial phenotype cell detected in peripheral blood will be considered malignant [23–25].

The Cellsearch system is composed of two devices. On one hand, the CellTracks AutoPrep for automatic sample preparation: it selects and detects CTCs by automated and standardized immunomagnetic enrichment of the cells. The separation of the cells is performed in two steps, the first one consisting in enrichment, the second one corresponding in detection. The selection was performed using ferrofluid particles coated with EpCAM antibodies. A permeabilization buffer and a fluorescent reagent were then added to the EpCAM positive cells. On the other hand, there is the CellTracks Analyzer II for analysis. This is a semi-automated fluorescence microscope connected to analysis software. This software allows the images obtained by 4 types of fluorescence filter to appear on a screen for the examiner to see. Finally, by observing the pictures, he will be able to confirm the presence of circulating tumor cells in the blood that was tested, and to count them. Tumor cells have their nucleus stained with DAPI (purple), the labeling around the nucleus by cytokeratins (green) and by CD45 [26].

We also used the reverse transcription-polymerase chain reaction (RT-PCR) technique in our study. This method is often used to characterize the expression of genes in various types of tissues or cells [27]. More specifically, the advanced cDNA synthesis kit iScript was used for this study. This kit is composed of nuclease free water, a reverse transcriptase enzyme and a reaction mixture with dNTPs and oligo(dt). Water control is used as a negative control, and no signal should be observed. The positive controls are the FaDu cells, before enrichment because they express the markers. If a signal is not observed after PCR, the assay cannot be validated. Extraction and quantification of RNA began with whole blood samples on PAXgene tubes. Then, transformation of RNA in cDNA by “Reverse Transcriptase” has been made. After making the reaction mixture, we used a thermal cycler for different successive steps. The first step consists in denaturation allowing the separation of the double chain of nucleic acids. It is followed by a hybridization step allowing the binding of the complementary bases of DNA.

In order to monitor the production of amplification products during each PCR cycle, we used fluorescent reporter molecules (SYBR Green). SYBR Green is fluorescent only when bound to dsDNA. The increase in fluorescence is proportional to the increase in double-stranded DNA. For this study, 9 markers were used: EphB4, Elf3, CEA, CK 18, CK19, Ep-CAM, EGFR, PVA, SCCA and GAPDH.

The evolution of each marker was studied by a mixed linear model to consider the repetition of measurements in the same patient. The fixed effect was time, the random effect was the patient. Two post-hoc analyses were carried out to compare the times two by two and a Bonferroni correction was used to correct the inflation of the alpha risk. This analysis was carried out either on the raw values, or on the values transformed by the logarithmic function, or on the values stored according to the Conover method.

An explanatory analysis was performed to study the influence of some of the clinical parameters (location, presence of emboli, lymph node status and infiltration) on the value of the markers. In order to do it, each marker at each time point was compared between the modalities of the clinical parameters. For the presence of emboli, lymph node status and infiltration, a Mann-Whitney test was used, and for localization it was a Kruskal-Wallis test. The significance threshold was set at 5%. Statistical analysis was performed using software version 9.3 (SAS, Cary, NC, USA).

### 2.3. Patients survival

With data obtained between 2014 and 2015, we monitored the survival of these 40 patients two years after their inclusion in the study. Thus, we wished to determine a link between the post-operative CTC rate and survival over two years. In a second step, we will evaluate whether the surgical procedure accelerated the cancer relapse by two years. Among the 40 patients, 14 died 2 years after inclusion. The time between inclusion and death of these patients ranged from 697 days to 23 days, with a median of 475 days. All patients received different post-surgical treatments. Among the 14 deceased, 5 had treatment with radiotherapy, 4 had with radiochemotherapy and 5 had no post-surgical treatment (often related to a patient’s refusal). In addition, in this sample of patients, 8 relapsed during treatment, 3 were treated with radiotherapy, 3 with radiochemotherapy and 2 had no postoperative treatment. It can also be noted that among the 8 patients who had a relapse, 5 died within 2 years after surgical resection (Table 2).

**Table 2:** Post-surgical treatment.

Treatment	Number of patients	Living patients	Deceased patients	Number of relapses
Radiotherapy	20	15	5	3
Radio-chemotherapy	10	6	4	3
Radio-chemotherapy + brachytherapy	1	1	0	0
Brachytherapy	1	1	0	0
No treatment	7	2	5	2

## 3. Results

### 3.1. Evolution of CTC rate

We wished to evaluate whether there was a correlation between the occurrence of death within 2 years and the evolution of the pre- and post-operative circulating tumor cell rate. Among the patients, 25 had cancer at stage III, and 15 at stage IV. There were 7 women and 33 men. We obtained usable results in 30 patients (Table 3). At the time of the study, the median age of these patients was 66 years (range 37-84 years). We were able to observe that out of 20 patients alive, 15 had a decrease in their CTC rate and 4 had an unchanged rate, and only one patient experienced an increase in this rate. Concerning the 10 patients who had a relapse, 6 had an increase in CTCs rate between pre-operative and post-operative sampling. Among these 6 patients, 4 died due to their cancer. 2 patients had a decrease in the CTC rate and relapsed. Nevertheless, it is noted that among these 2 patients, one had a high initial rate, which can potentially explain this therapeutic failure. Finally, 2 patients who relapsed had an unchanged rate during the study.

**Table 3:** Evolution of the CTCs rate compared to patient survival.

Evolution of the rate of CTCs	Number of patients	Living patients	Deceased patients	Relapses
Decrease	17	15	0	2
Unchanged	6	4	0	2
Increase	7	1	4	4
Total	30	20	4	10

### 3.2. Results with CellSearch and RT-PCR method

**Fig 1:**
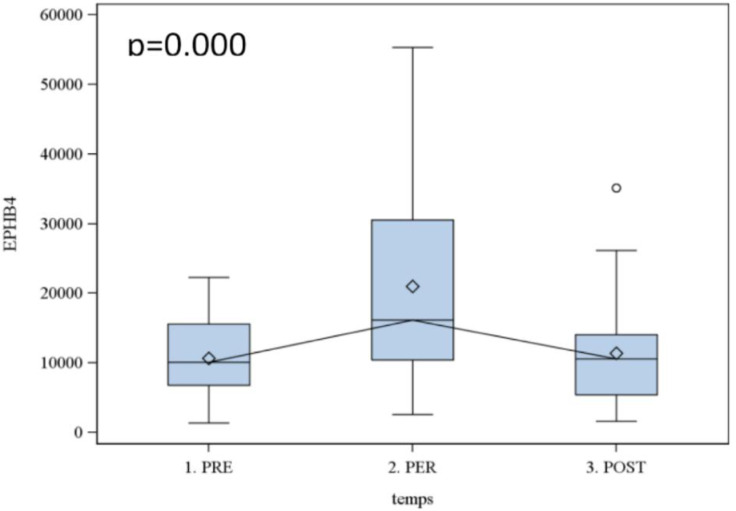
Box plot results for EphB4.

**Fig 2:**
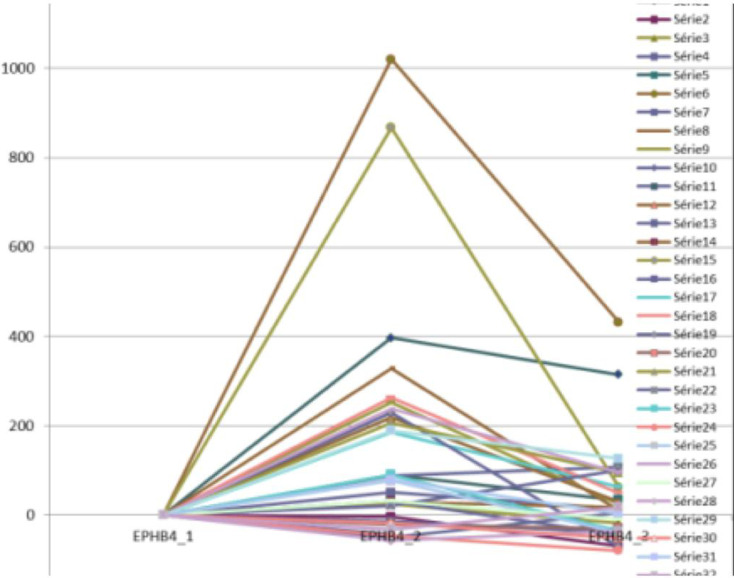
EphB4 expression for each patient.

The RT-PCR method gave significant results (p <  0.05) for EphB4, CEA, CK 18 and Ep-CAM. For these markers, the surgical procedure shows an impact: two hours after surgery, the variation rate of markers is higher (meaning an augmentation of the CTC); and 7 to 8 days after it regulates in the blood circulation. For example, the box plot for EphB4 [28] shows the impact of surgery, with a 1.5-fold increase in marker expression 2 hours after the surgery’s beginning, returning to the baseline (pre-operative) lever after 7 days (Fig 1). Individual variations in EphB4 expression for each patient studied are depicted (Fig 2). We can notice significant fluctuations in EphB4 expression, possibly indicating the presence of a distinct subpopulation of tumor cells. However, the other markers tested were not significantly expressed.

In addition, the results obtained with the CellSearch system using the Epithelial kit appeared non-significant (p > 0.05), while the expression of EGFR by this system was statistically significant (p=0.01). Moreover, a significant decrease in EGFR expression (p<0.05) was observed in all patients included in the study. This decrease occurred within two hours after surgery and remained stable seven days after surgery.

**Fig 3:**
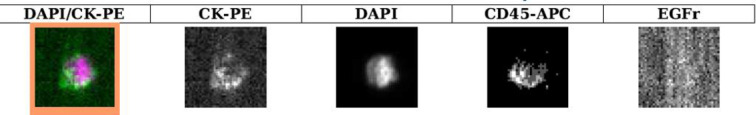
CTCs images obtained with CellSearch.

CTCs are detected in fluorescence after labeling the nucleus (DAPI, blue fluorescence), immunolabeling of CK8, 18, 19 (green fluorescence) and of the CD45 antigen (red fluorescence). They have a round morphology, a CK+ cytoplasm and do not express the CD45 antigen. The results are expressed as the number of CTCs/7.5mL of blood. Below, there is a CTC with nucleus stained red, with EGFR well expressed and stained green (Fig 3). The result is negative for CD 45, and it confirms the tumor nature. It is also positive for CK markers (8-18-19).

## 4. Discussion

This study allowed us to identify the fact that the variation rate of some markers was specific for head and neck cancers, and because of that, it could be used in the following part of the project. The RT-PCR results are promising, and an optimization of the protocols is necessary to obtain a better sensibility of detection and the establishment of a decisional tree. Indeed, the same types of studies that were conducted on lungs, esophagus, or even breast cancer have concluded on the establishment of a decisional tree regarding the markers’ positivity. By combining the presence of RNAm of CEA and of Survivin (member of the apoptosis inhibitors’ family), revealed by RT-PCR in blood samples from patients with esophagus cancer after surgery, Liu Z. and al [29] proved that it could predict the risk of developing micrometastasis. In case of lung cancer, it has been demonstrated that the combination of three markers (TSA-9, pre-proGRP and KRT-19) in the CTC detection increased the positivity of 84,3% [30]. At last, the simultaneous detection of Survivin, hTERT and hMAMA ARN transcripts in breast cancer allowed a sensitivity of CTC detection of 70,2% [31].

With a simple blood test, regardless of the method used, the number of CTCs by blood unit can be determined. It has been demonstrated that this number grows with the stage of the tumor. If the number of circulating tumor cells is inferior to 5/mL in blood, the prognosis is considered good regarding the risk of relapse. On the contrary, if the number is superior to 5, the prognosis is considered bad. Quantitative variation of this number between the diagnosis and the first cycle of chemotherapy allows the medical team to predict the response to the treatment, earlier than the radiologic evaluation can [32]. Moreover, studying the CTCs after isolation allows the doctors to predict which treatment will be the most efficient on metastasis. Thanks to that fact, treatments could be adapted and more useful [33]. A limit of 5 circulating tumor cells/7,5mL of blood has been set: if a patient shows more than 5 cells, the probability of them developing metastatic cancer is increased [17]. In our case, and based on the results, we cannot conclude definitively. However, we can notice a trend as most patients in remission had a decrease in their post-operative CTCs, and patients in relapse had an increase in their CTCs.

The markers’ choice for detection revealed itself to be tough since head and neck epidermoid cancer have numerous markers in common with other cell types of cancer. As explained earlier, the search for circulating tumor cells in other cancers (breast, colon, prostate, etc.) has been well targeted and studied, and is a part of the patient’s care during diagnosis and treatment. Yet, in head and neck epidermoid cancer there is no defined marker. Overall, the first results showed that the RT-PCR or CellSearch both were specific and sensitive methods of detection in epidermoid head and neck cancer.

Using different methods of detection represents the first approach in this project regarding CTCs detection in epidermoid head and neck cancers. Regarding CellSearch, there is for now no specific kit for head and neck cancer but using an anti-EGFR antibody in addition to the epithelial kit makes the detection a possible option. This method has already been used, especially in breast cancer, and has proven its efficiency [34,35]. This method has an important predictability, yet it is not suitable for the actual commercial kit, in breast, colon or prostate cancer. We have adapted our research to detection with CellSearch: in addition, the EGFR marker was used, and results are very promising. The use of this method is more practical but only qualitative. During the CellSearch analysis, the EGFR marker decreased significantly. Such a result could be linked with the total removal of the tumoral mass since EGFR is a component of practically every tumoral cell. A total ablation could cause a total decrease of CTCs.

The RT-PCR real time method is, on the contrary, a quantitative method. Results obtained in our protocol have been analyzed in a statistical way, as well as regarding the particularity of each individually. Before the analysis, phenomena linked to the surgical act must be considered: if the surgical act concerns a total removal of the tumor (tumoral mass and nodal one), then the logical consequence would be a diminution or a disappearance of the CTCs phenomenon. Opposed to that, if mechanical traumatism occurs during the surgical procedure, CTCs are supposed to increase. Now, considering the results in their globality, if 4 markers increased regarding the number of tumor cells already there, it means that they probably represent the tumoral phenotype of the cells freed during the surgery. These markers representing the CTCs could mask the presence of different tumoral underpopulation. In addition, because of their significance, their potential biological activity could be discussed.

Considering individual aspects of the results, the notion of the patient’s individuality could also be discussed. Indeed, the phenotype and amount of circulating tumor cells can depend on the patient: in some cases, all the markers increased after the surgery, in others only a few were increased (probably suggesting a small amount of tumor cells under-populations). When little variation, or no tumor cell variation, is observed, it can be considered that the surgical procedure had no impact on the cells’ liberation. When a diminution of the markers is observed, again it can be supposed that the tumoral mass was globally removed, as it was mentioned regarding EGFR. In the end, an augmentation of the time of some markers can be considered. Questions of circulating tumor cells multiplication, and demarginalization of sleeping cells from a location where they accumulated can be highlighted.

This study was based on the follow-up of 30 patients 2 years after their surgery and showed that 95% of the living patients had decreased or unchanged CTCs. In addition, among patients who died and/or relapsed, 60% of them had an increase in their CTCs post-surgery. We have noticed that 85% of the patients who had an increased CTCs level between the preoperative and postoperative period died and/or had a cancer relapse within 2 years after surgery. Thus, we can suggest that an increase in CTCs is an indicator of poor prognosis. Also, we have noticed that every patient who had no CTCs in three blood samples remained alive and without relapsing within 2 years post-operatively. This study has thus highlighted the promising character of the study of CTCs levels in patient follow-up, and it would be interesting to study this phenomenon on a larger patient sample. Indeed, there is a limited amount of published research on the influence of circulating tumor cells in head and neck cancer. Nevertheless, Grisanti and al. [36] were also able to demonstrate on 53 patients that CTC detection holds significant prognostic value and has the potential to predict treatment efficacy.

This study ran into no limitations, except for one patient who had to leave the study before the end.

## 5. Conclusions

This study consisted of an analysis of biological factors aimed at identifying the presence of tumor cells circulating in the blood before, during and after surgery. This protocol demonstrated statistically significant results of variation in circulating cell populations. The two detection methods used (RT-PCR and CellSearch) allow us to validate with concordant results, quantitatively and qualitatively, our working hypothesis. The different techniques used in this project represent a first approach to the subject of the detection and selection of CTCs from head and neck cancer. On top of that, we were able to demonstrate that an increasing rate of CTCs after surgery could potentially be an indicator of poor prognosis for the patient, though a larger sample of patient is necessary. Studies are promising, allowing the possibility to detect relapses earlier in the care of the patient. In the future, it could be interesting to conduct research by circulating DNA for example.

## Supporting information

S1 File Markers charts per patient. Charts showing the evolution of the markers depending on the time. (PDF)

S2 File Patients’ samples. Report on the patients’ samples. (PDF)

S3 File Structured results. Structured results of CTCs in patients. (PDF)
