# Supplementary figures and images for: Study of the survival of patients with head and neck cancer in relation to Circulating Tumor Cells (CTCs)
Source: PLoS One. 2025 Apr 1;20(4):e0320485. doi: 10.1371/journal.pone.0320485 (PMC11960953; doi:10.1371/journal.pone.0320485)

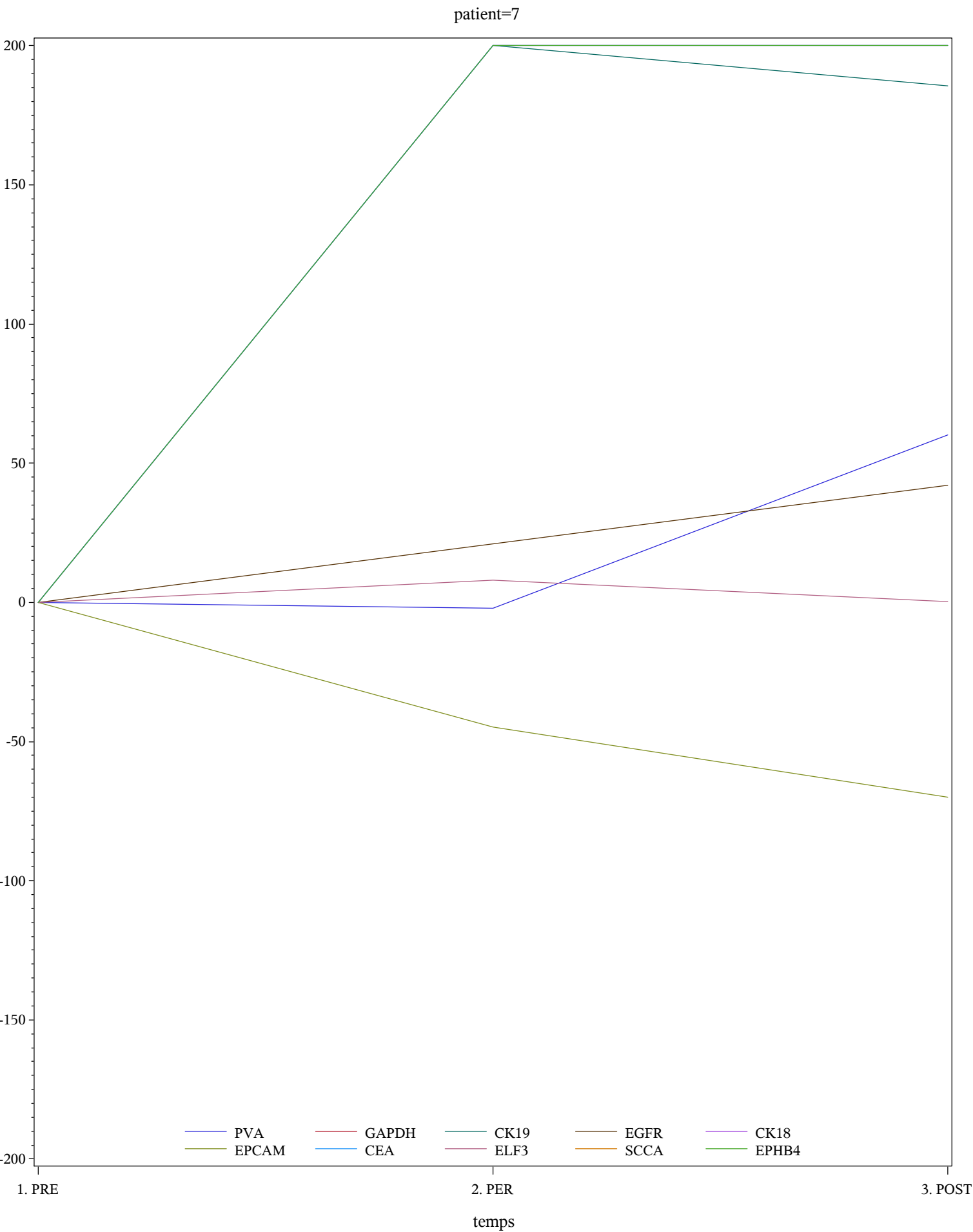

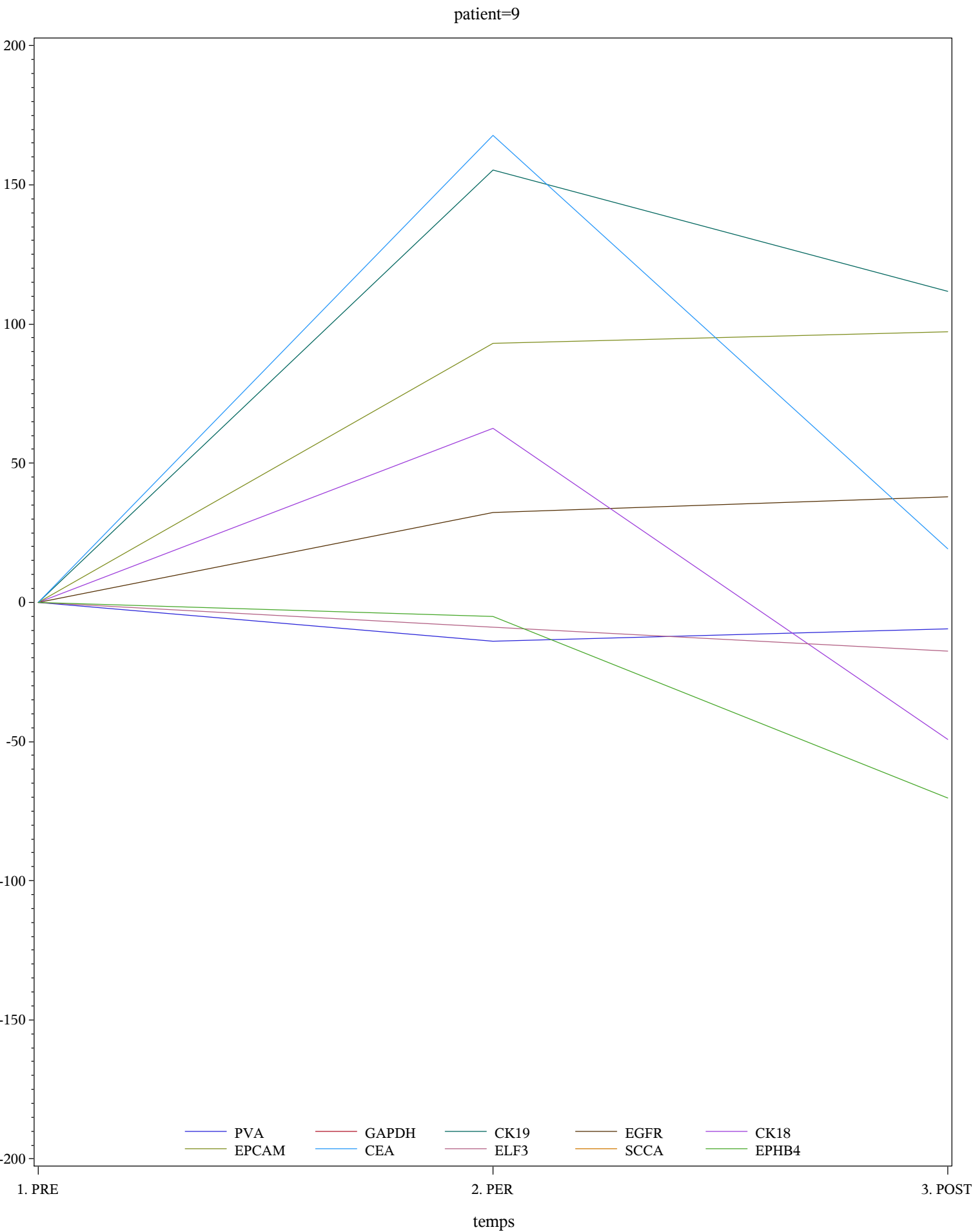

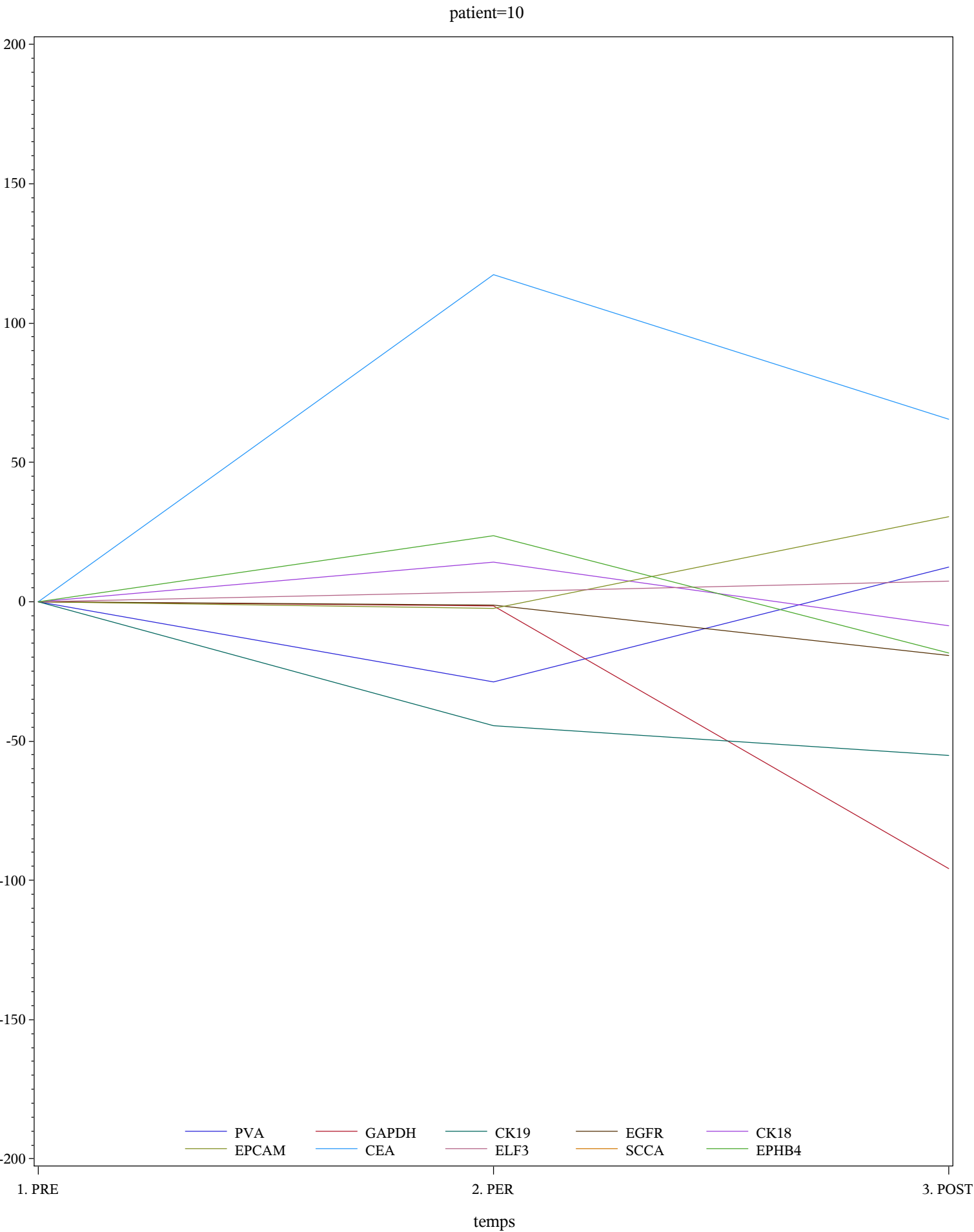

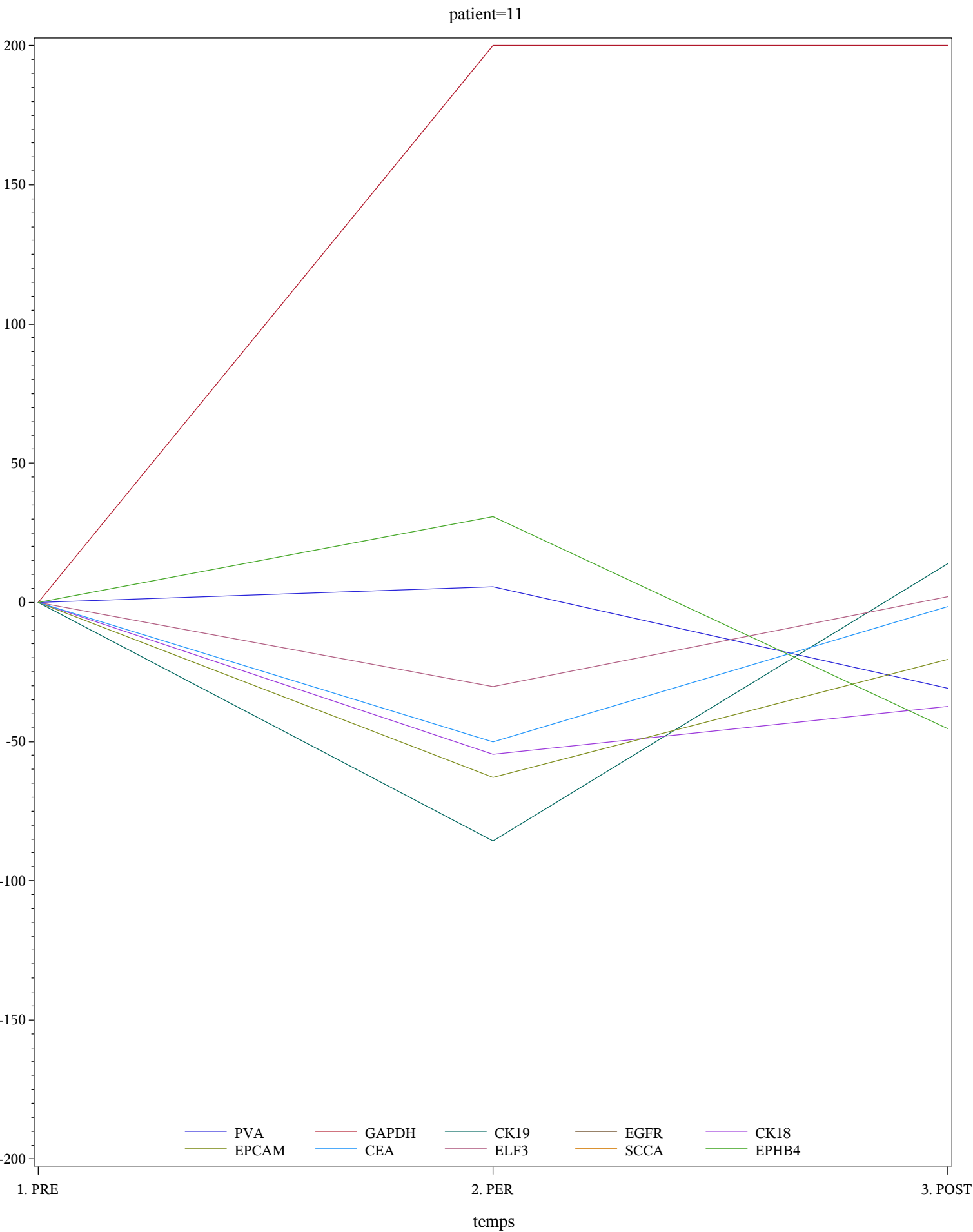

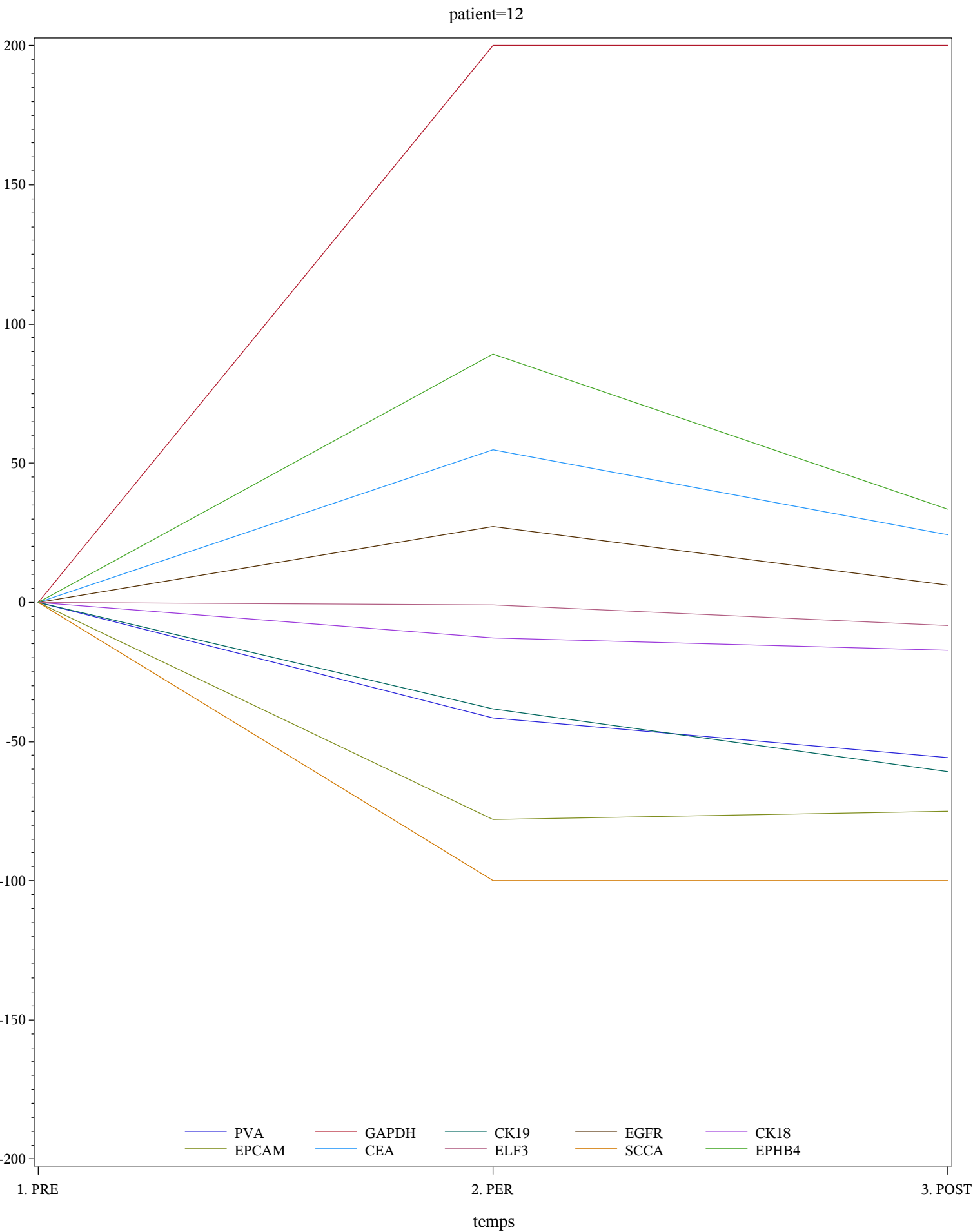

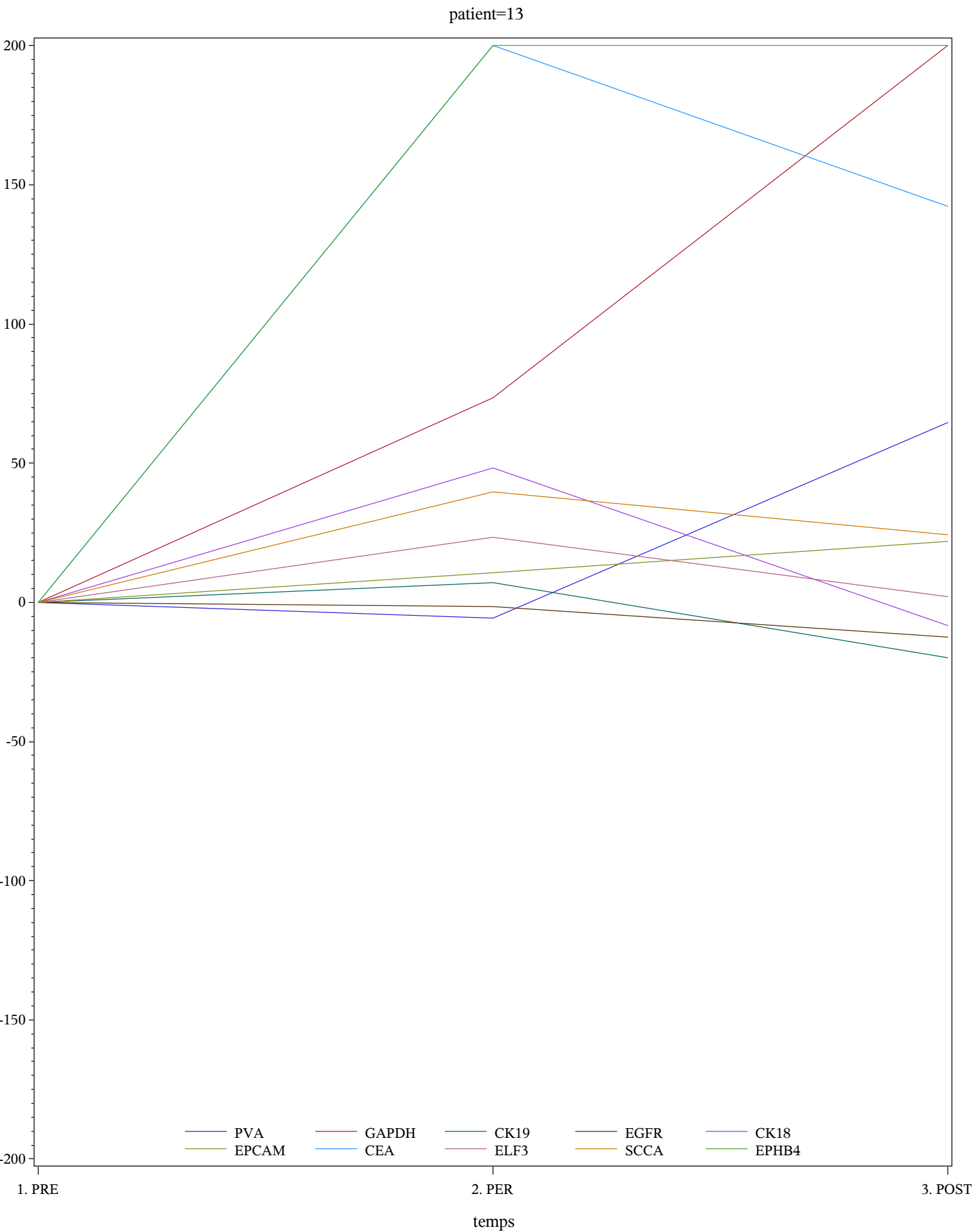

patient=14

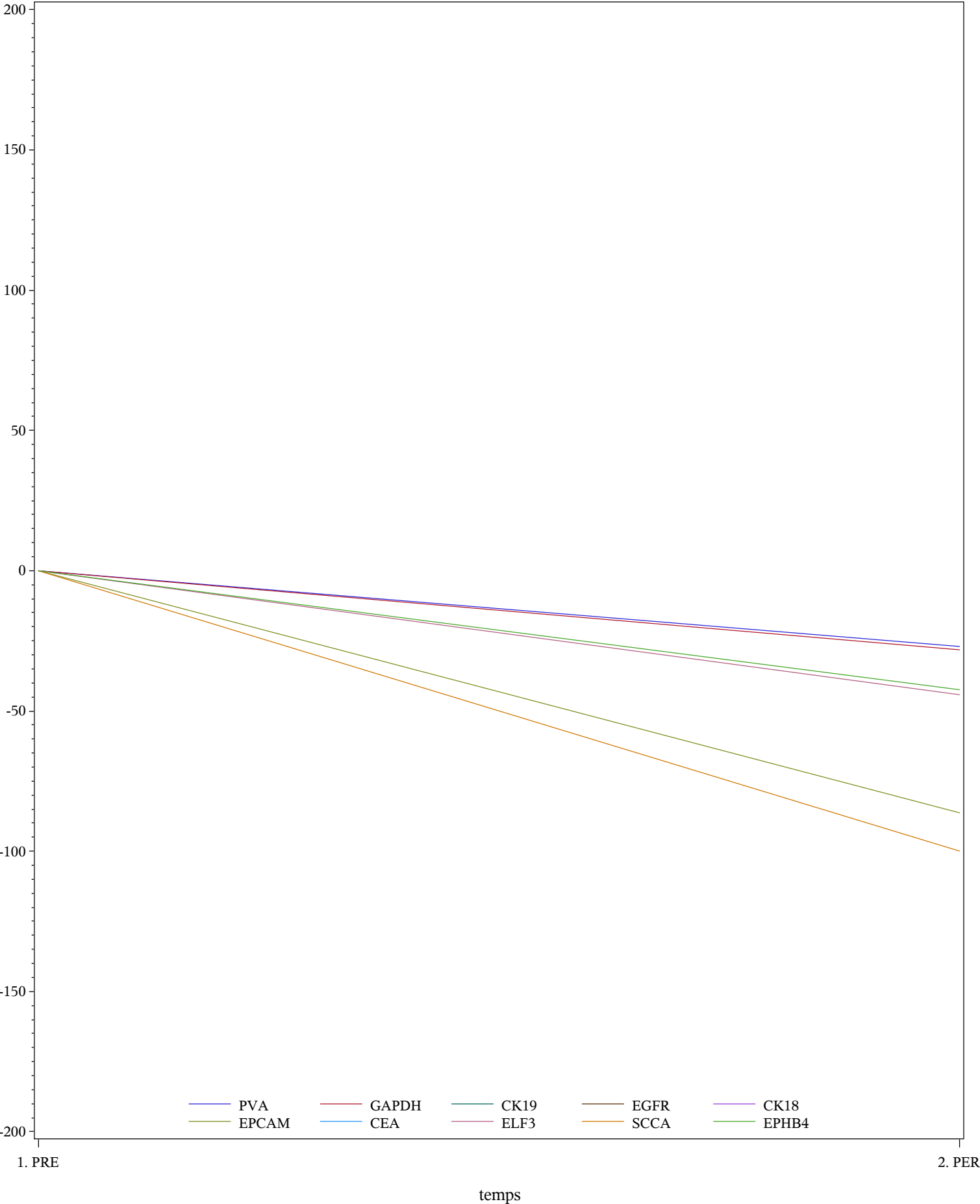

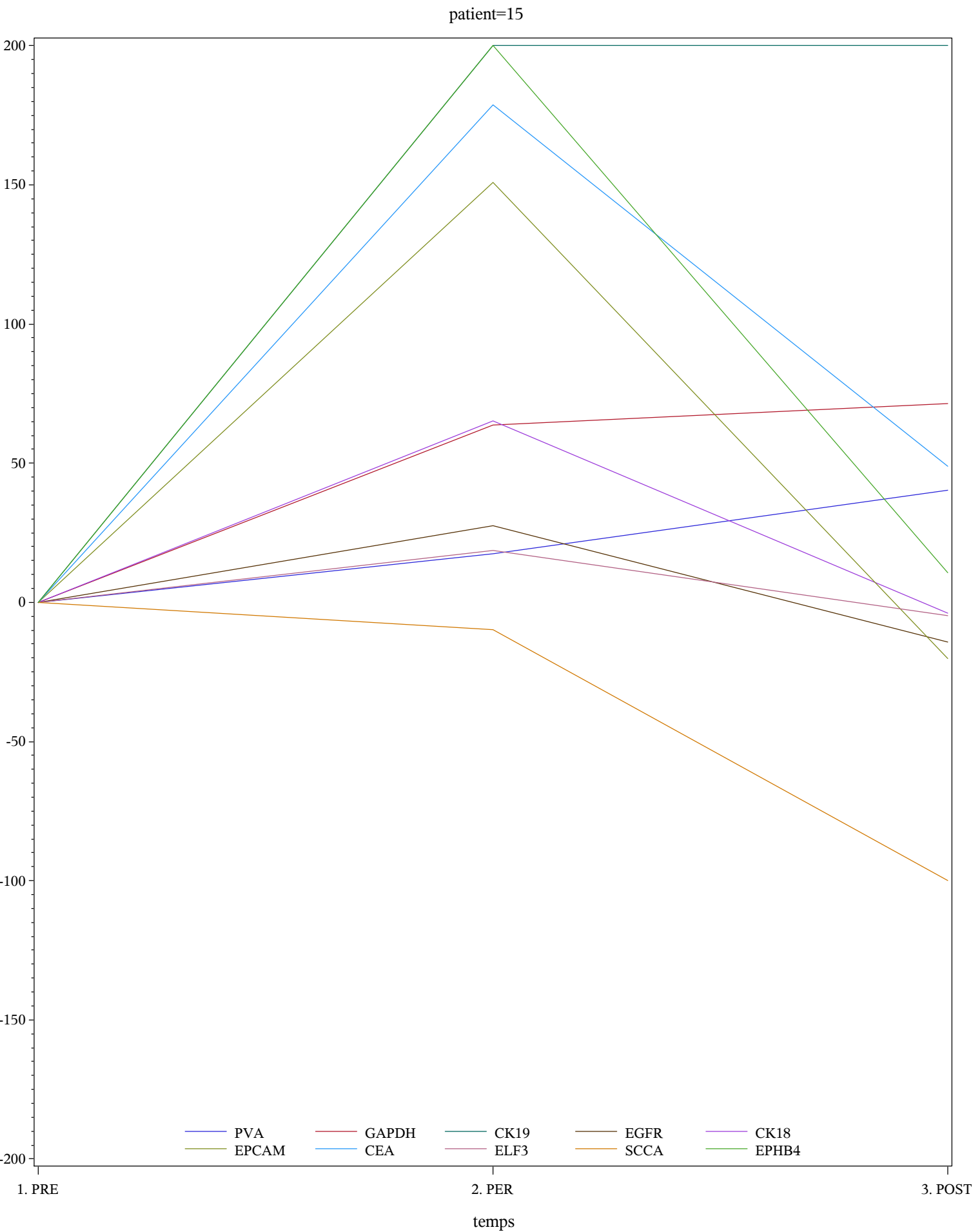

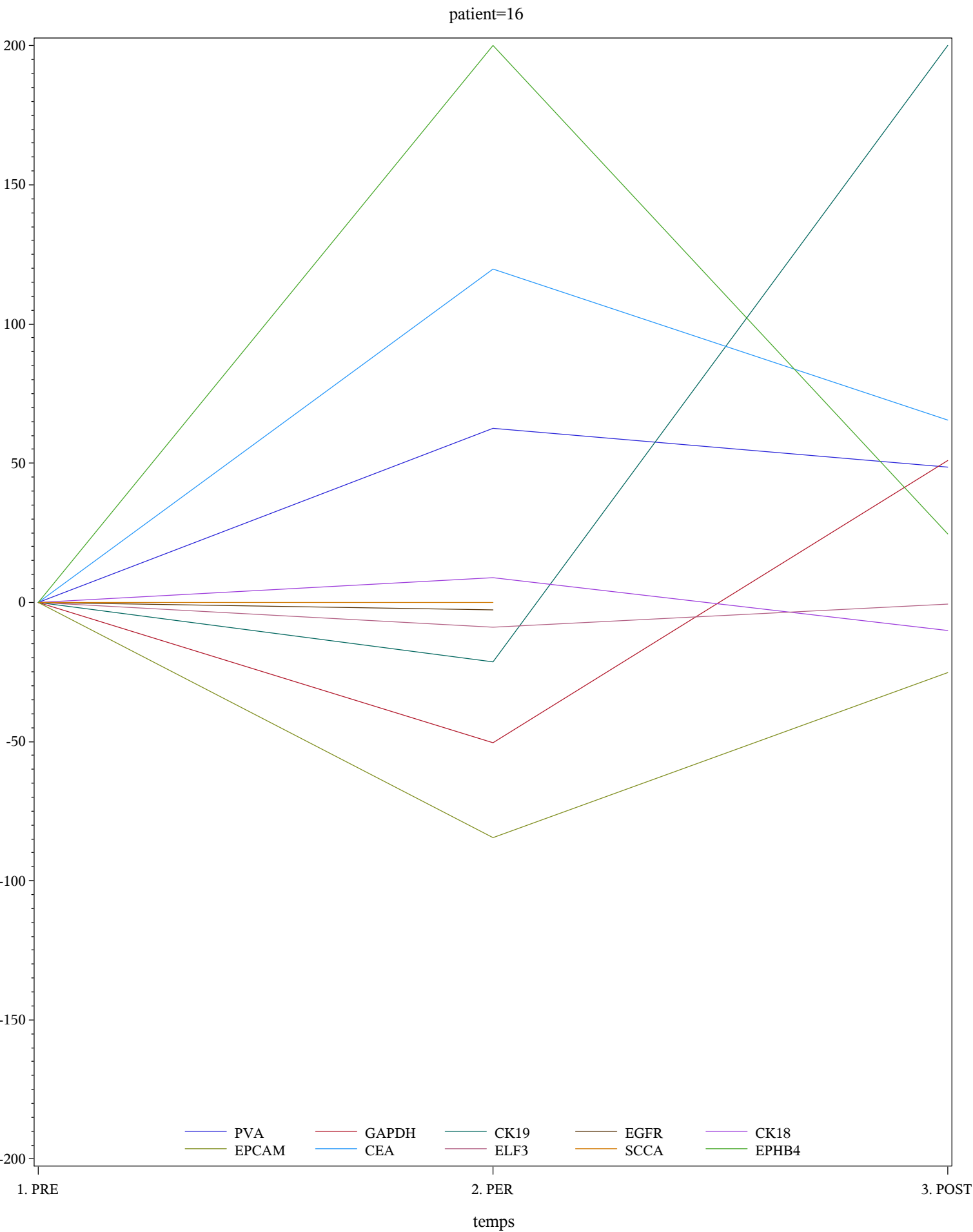

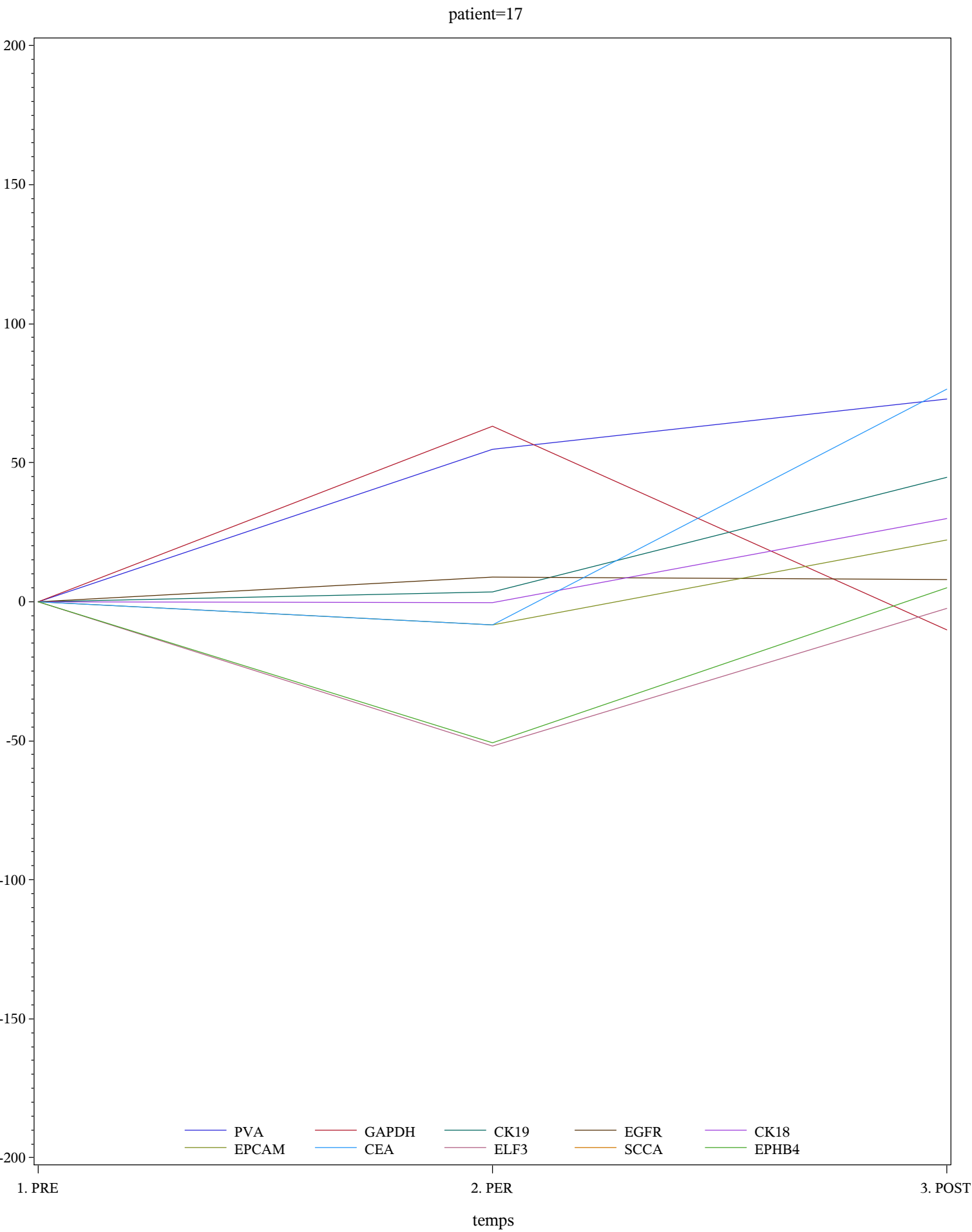

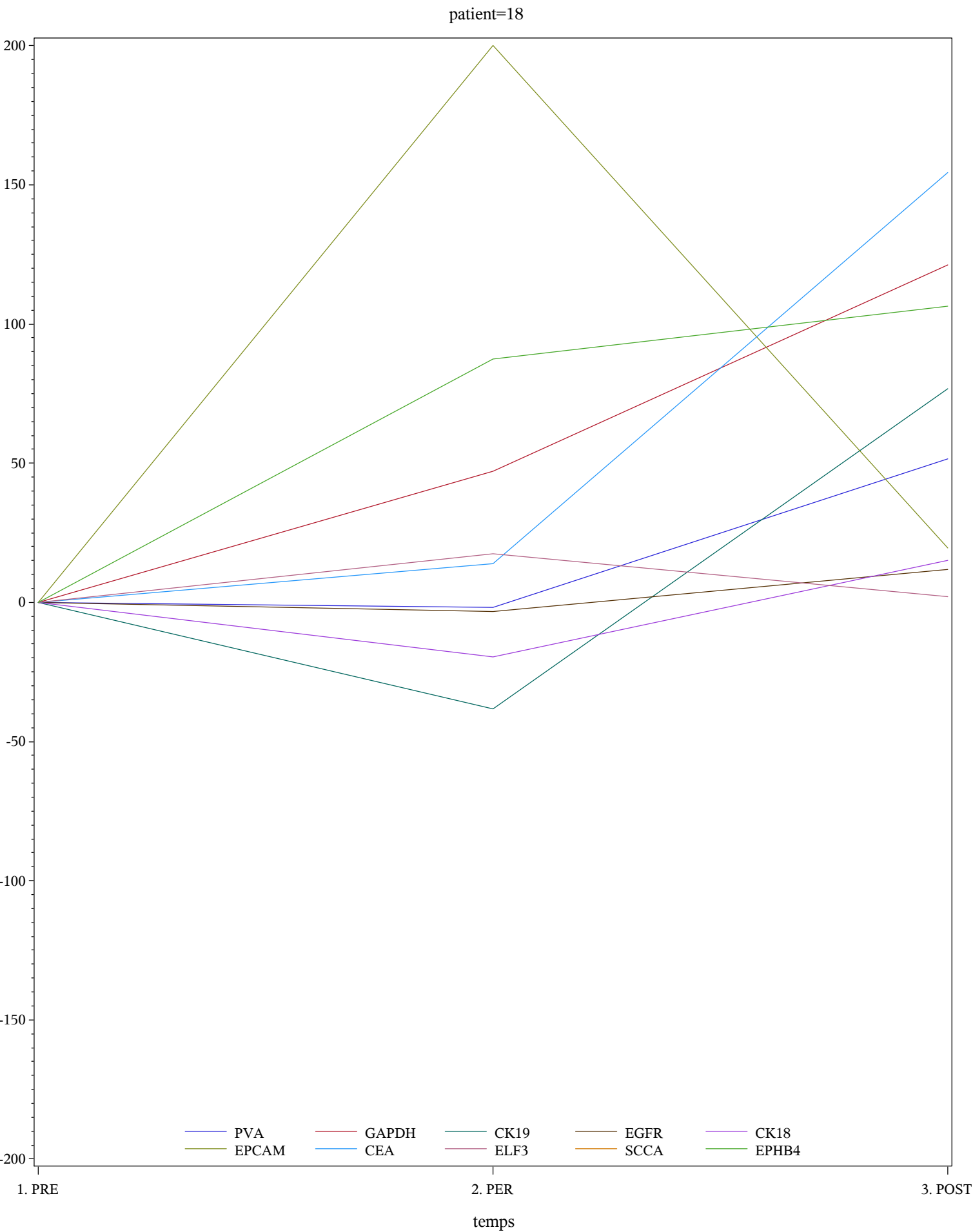

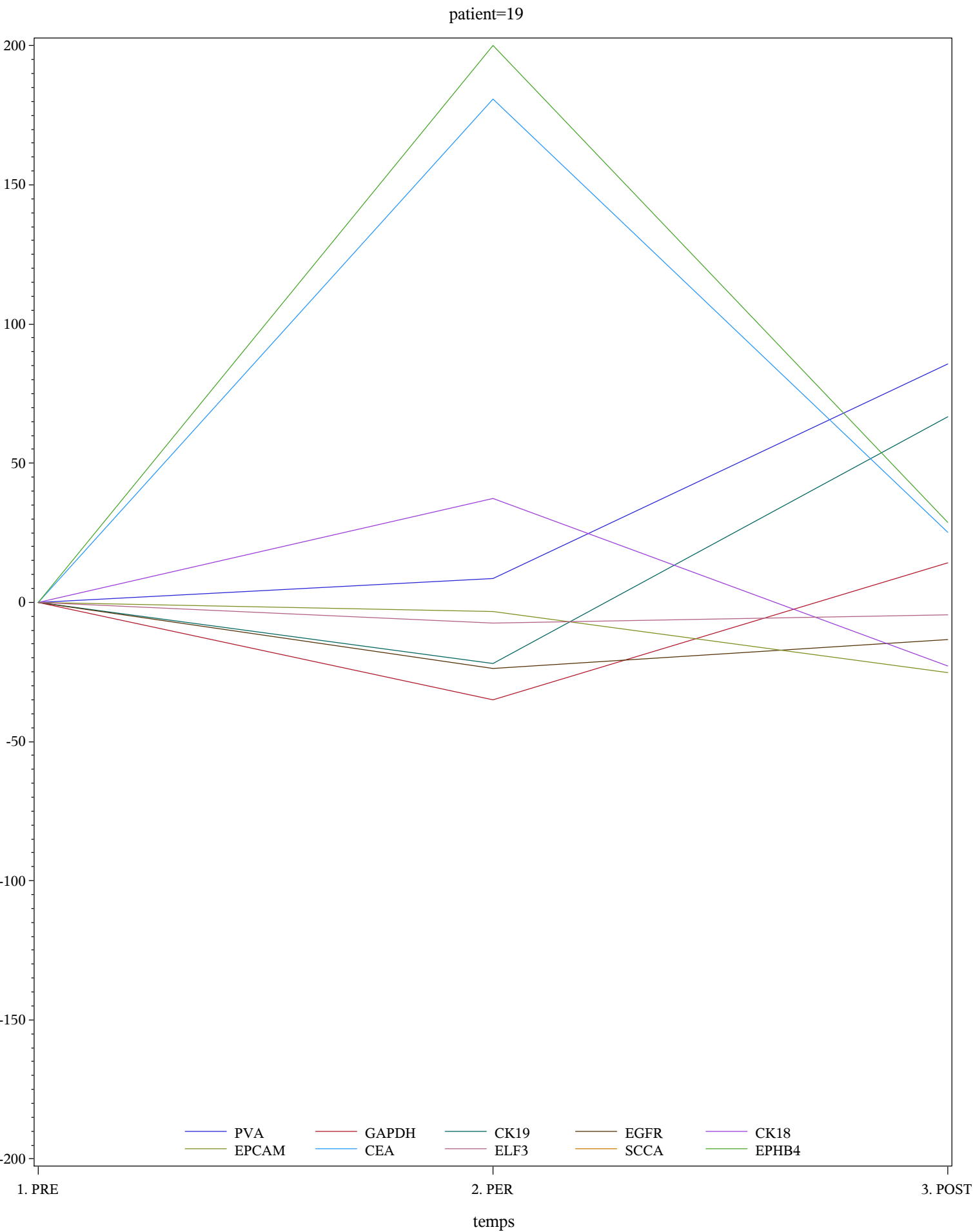

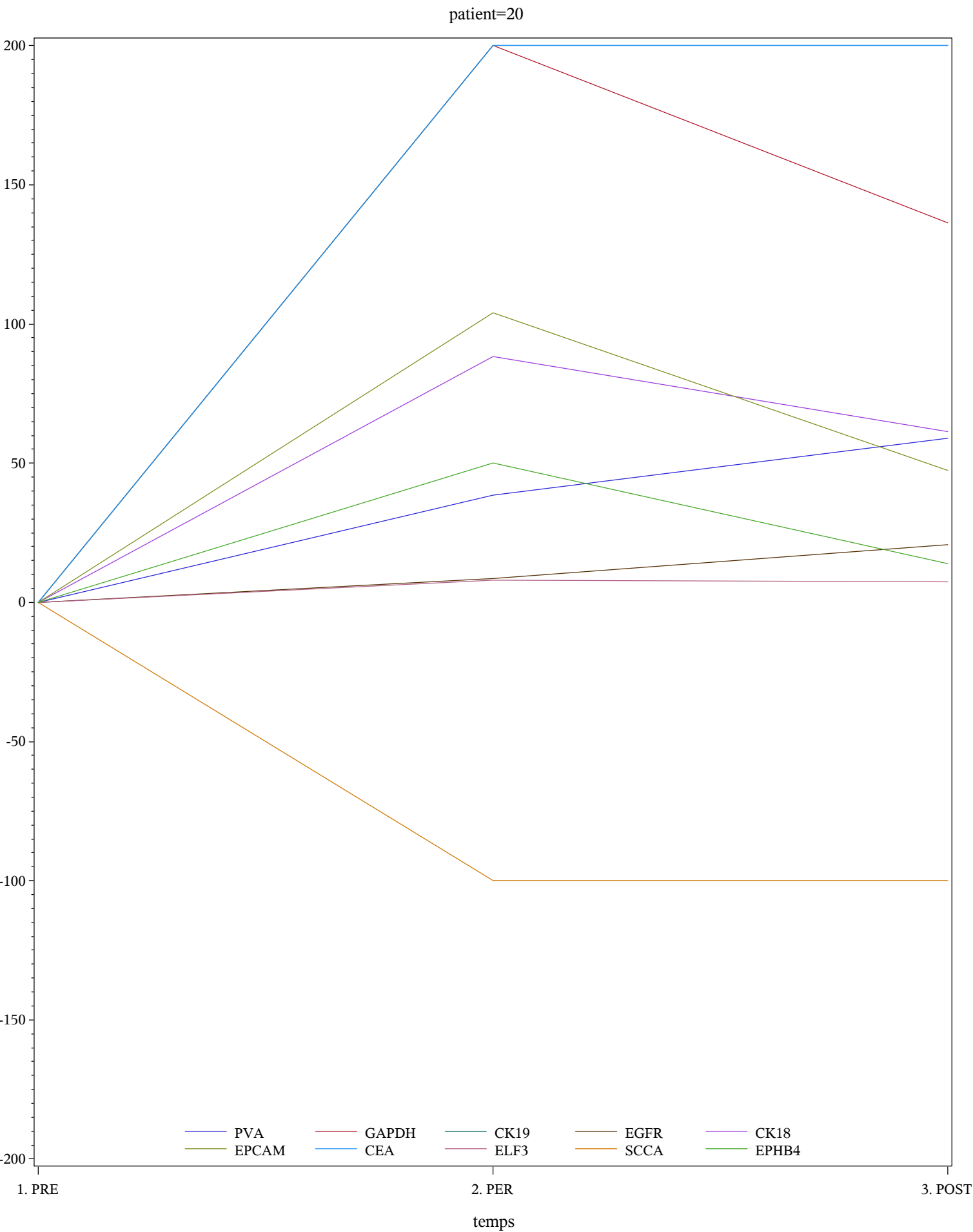

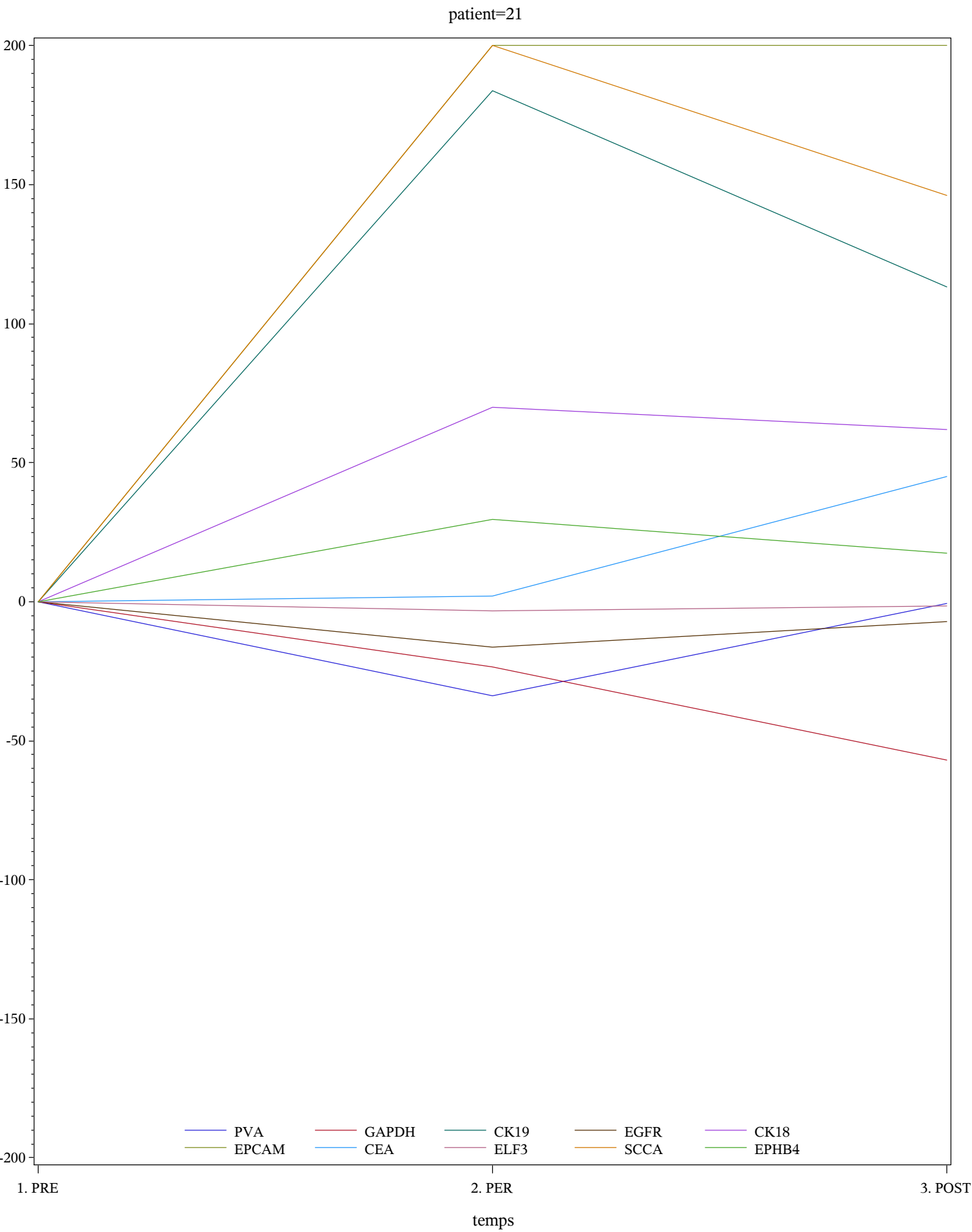

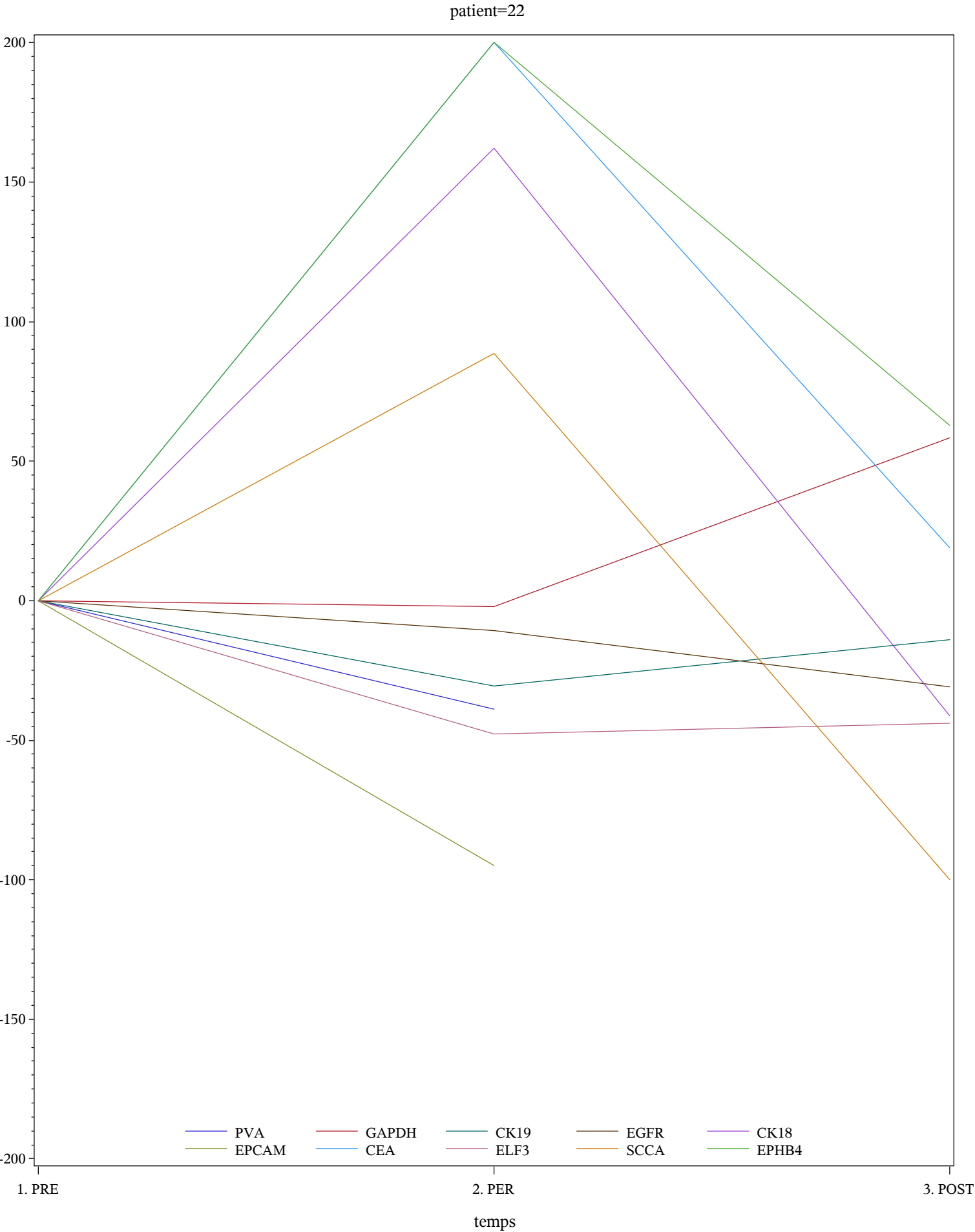

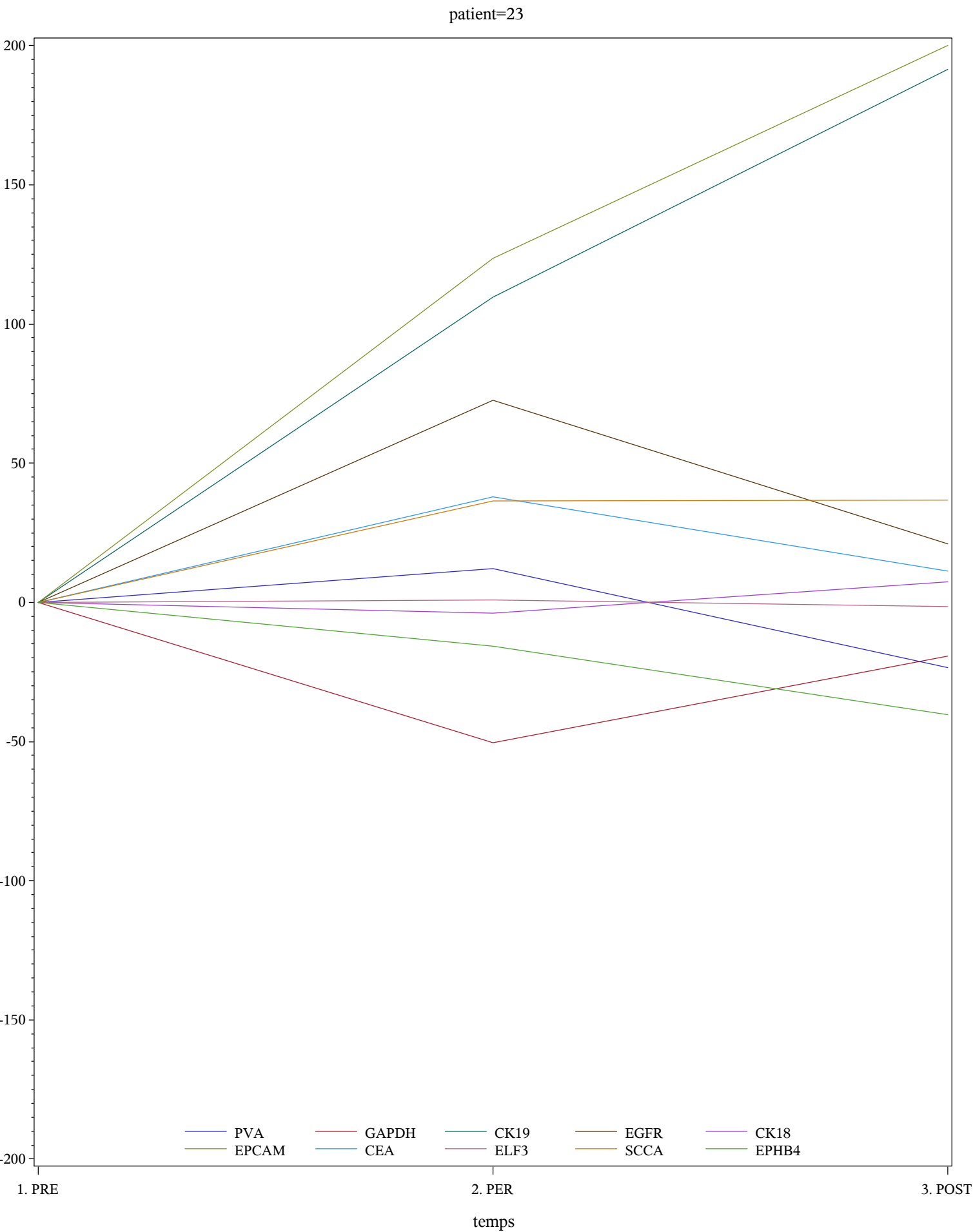

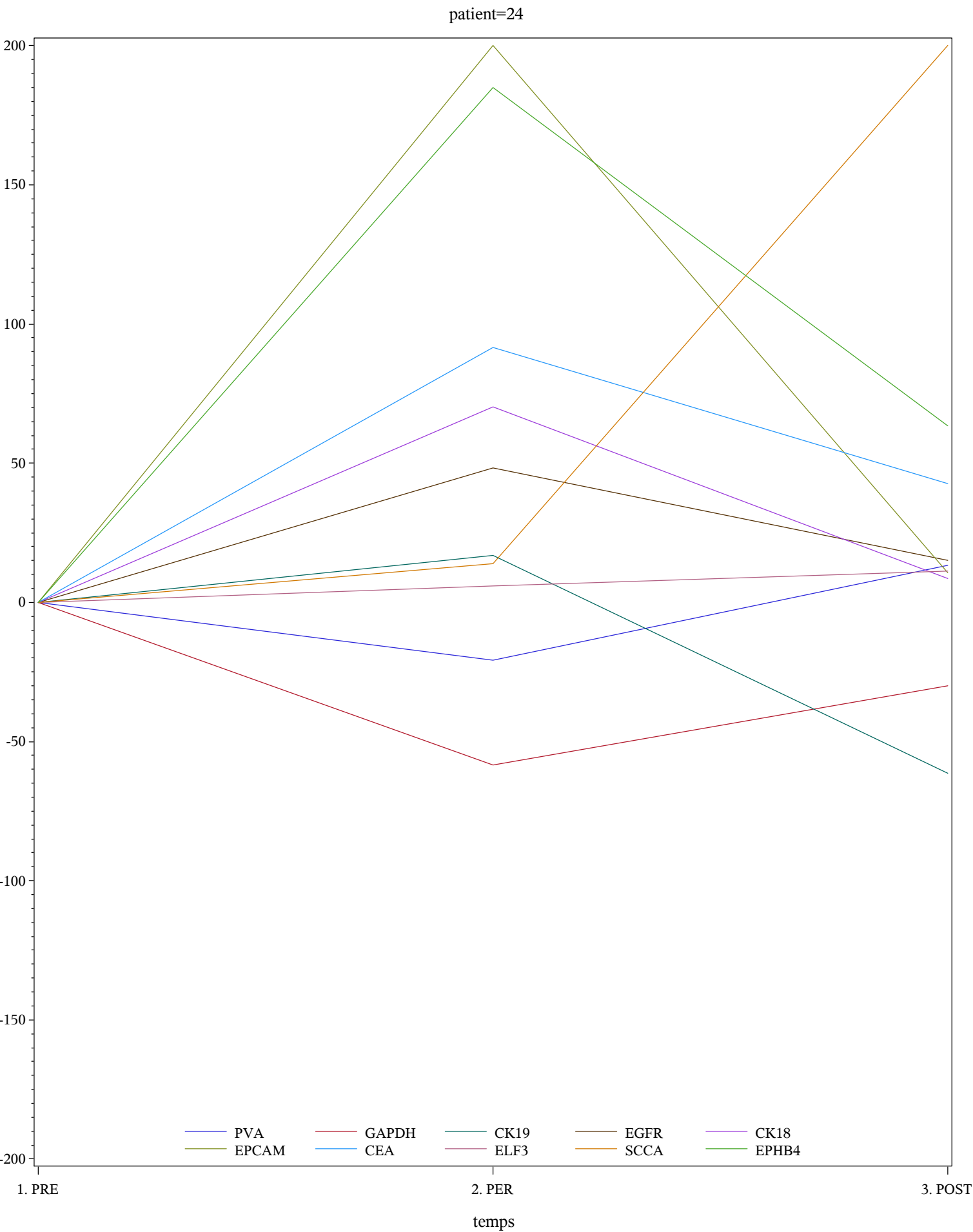

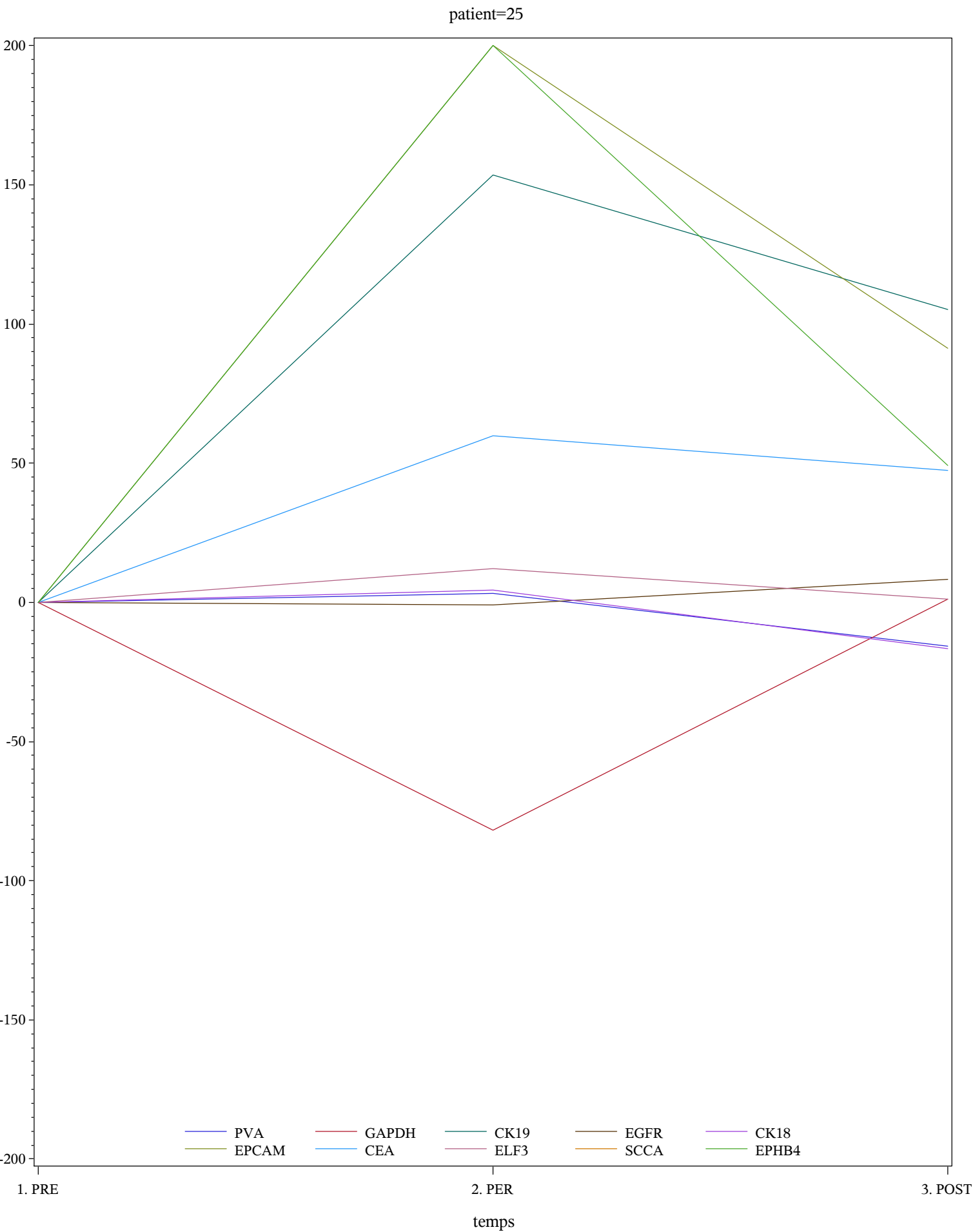

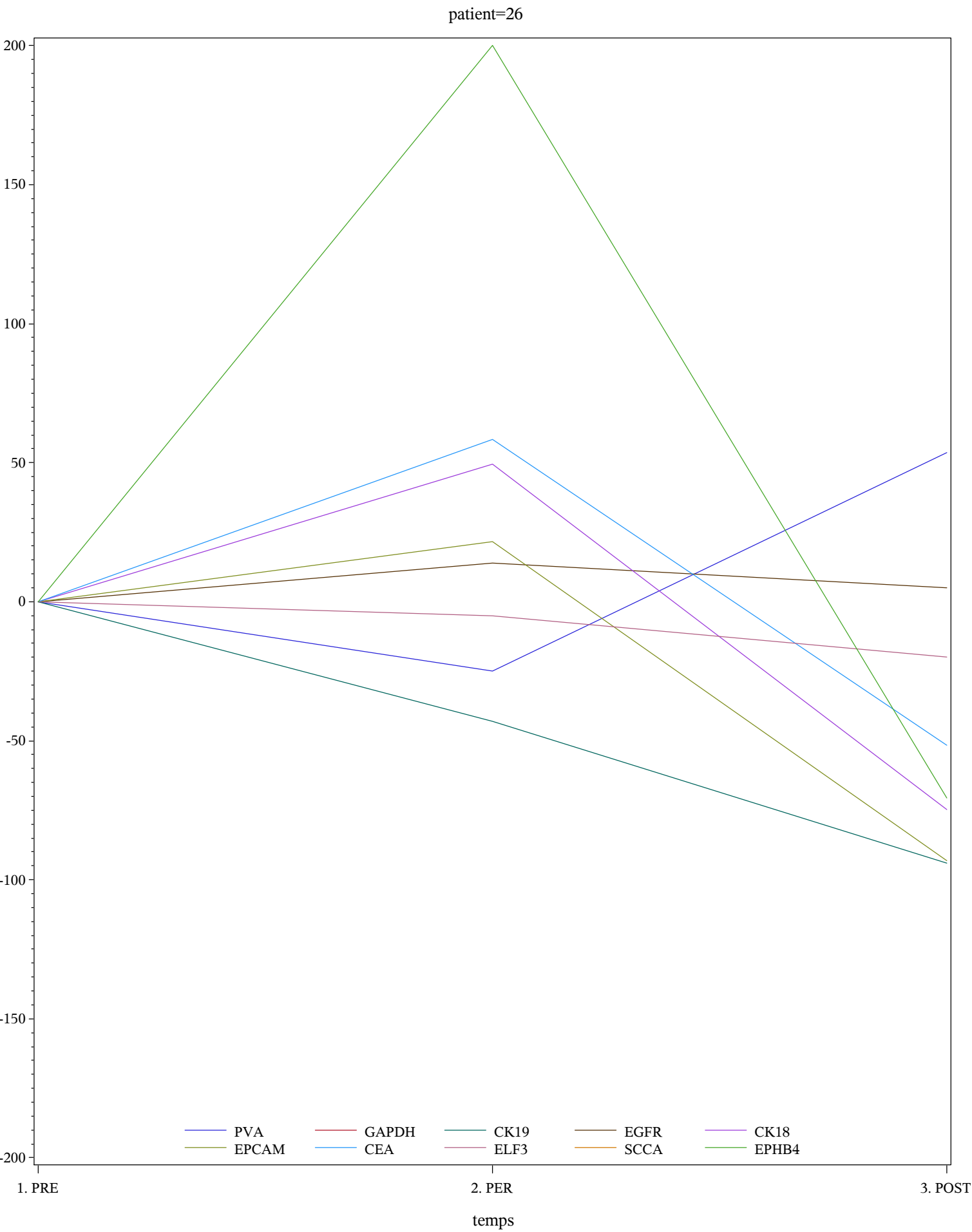

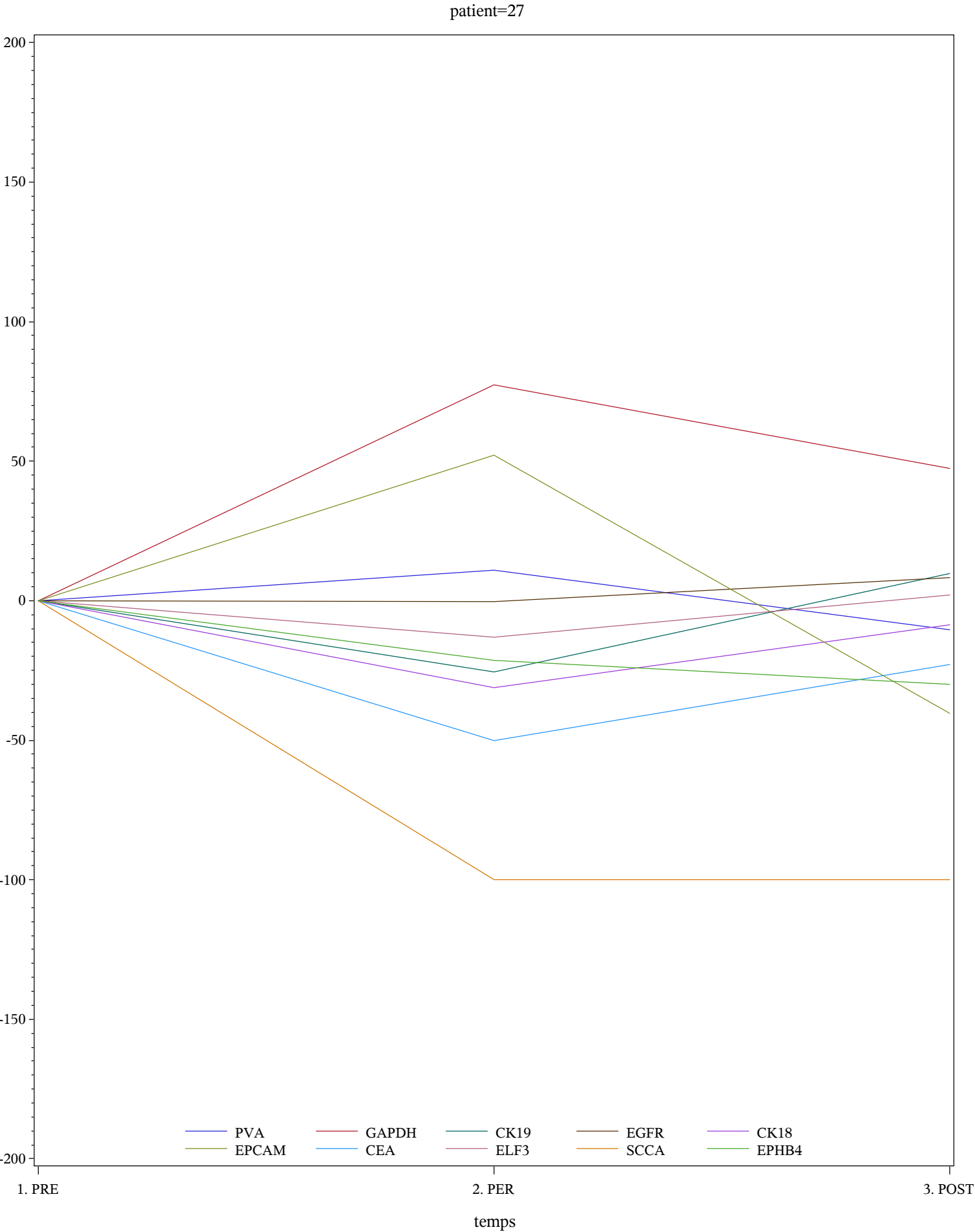

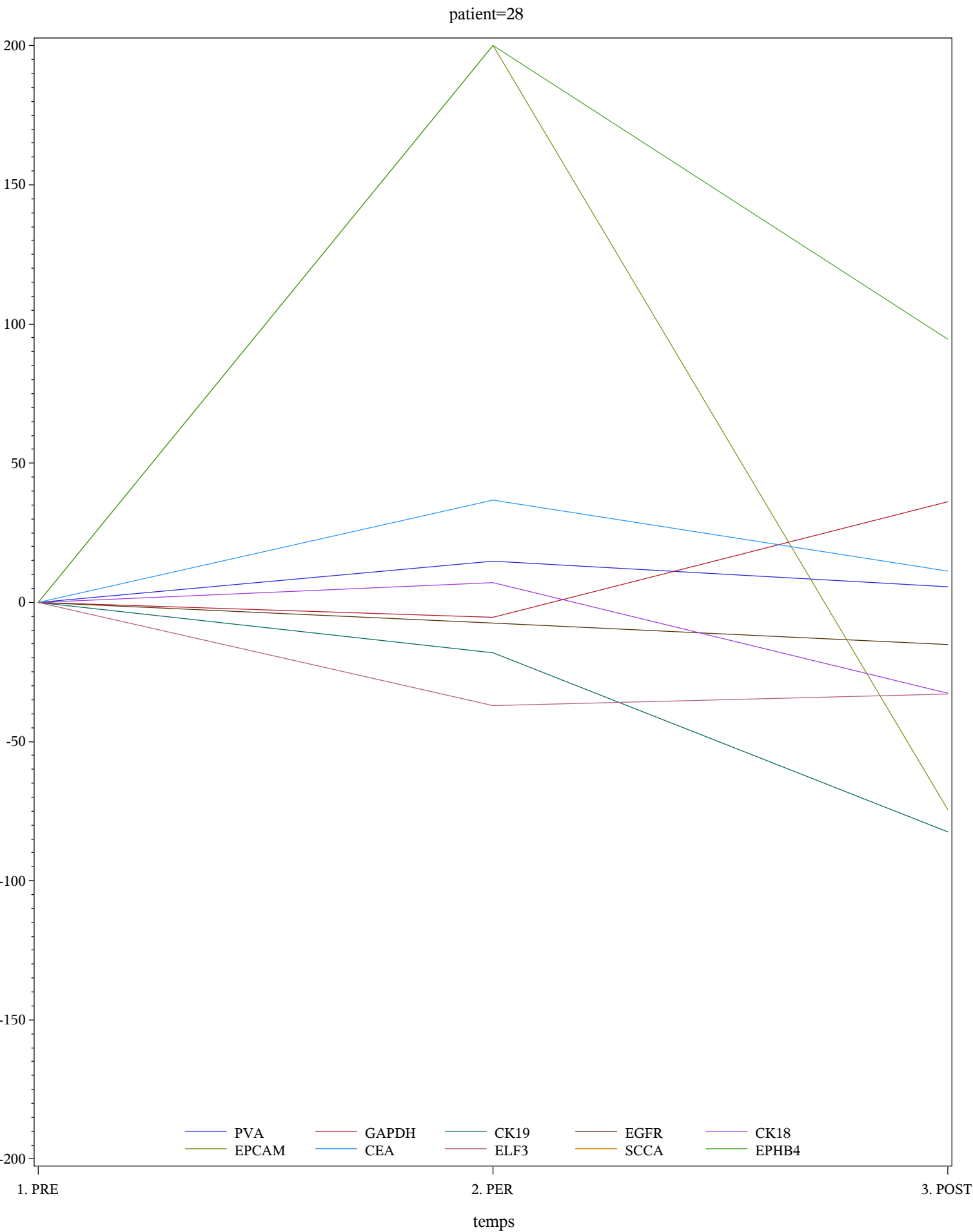

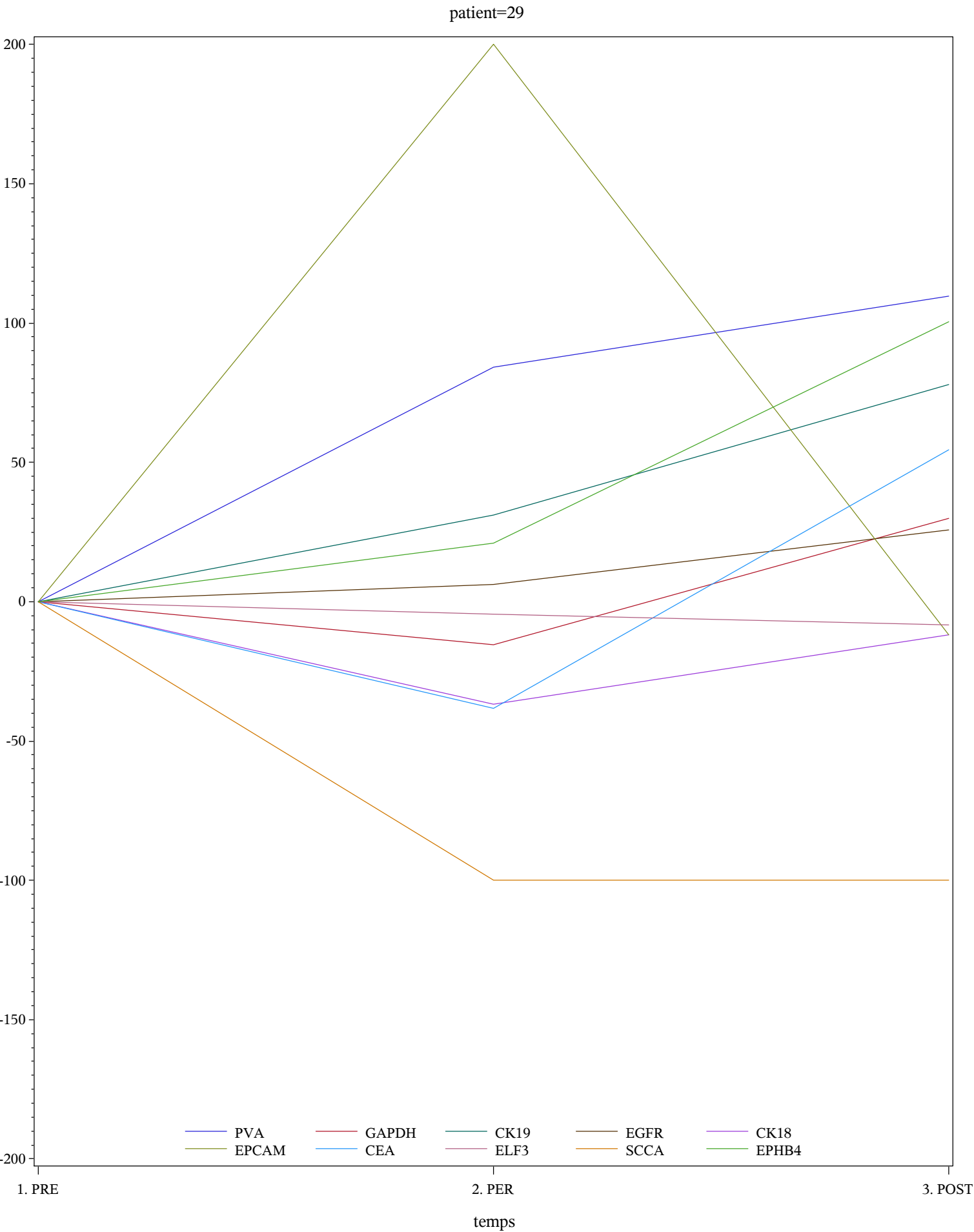

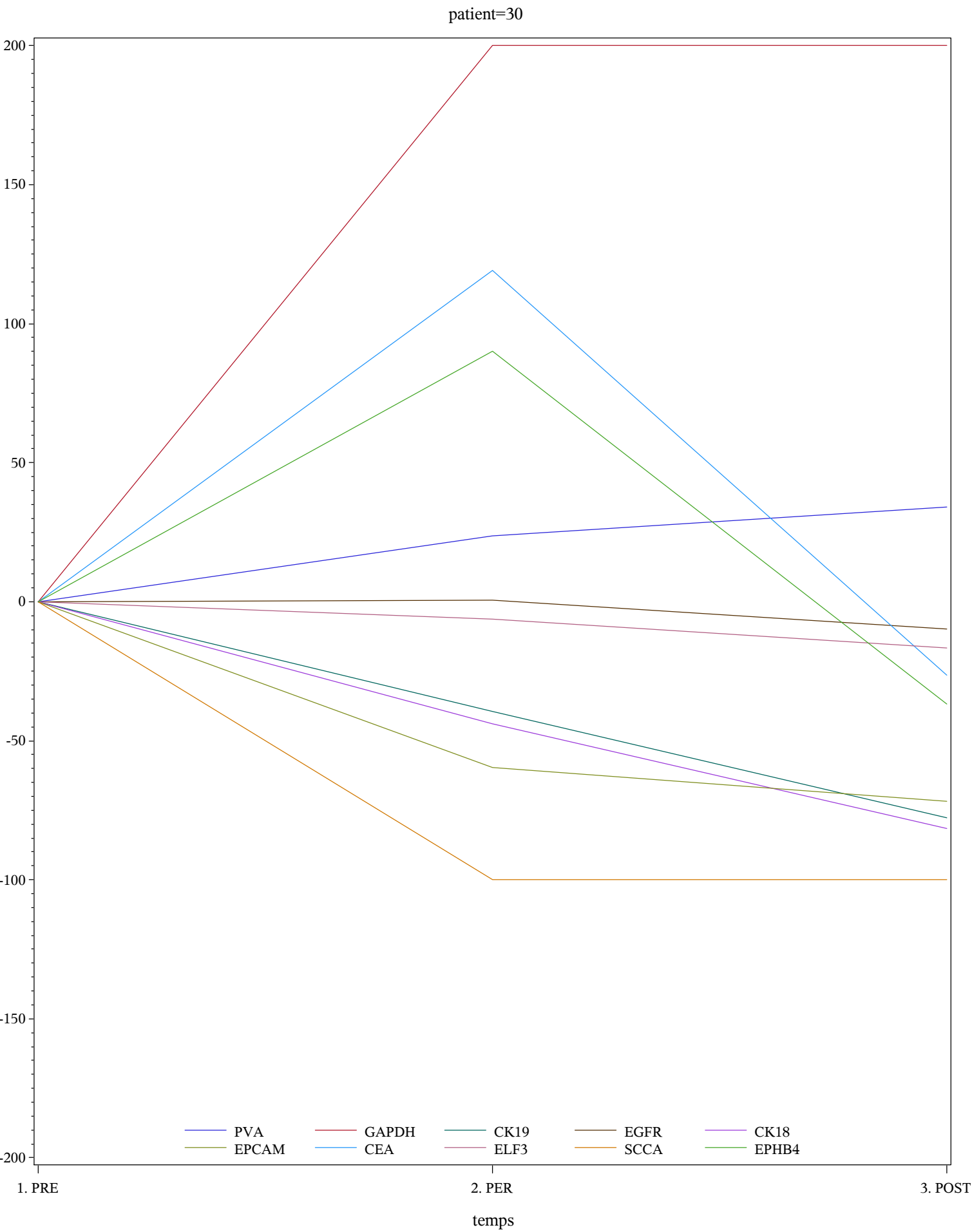

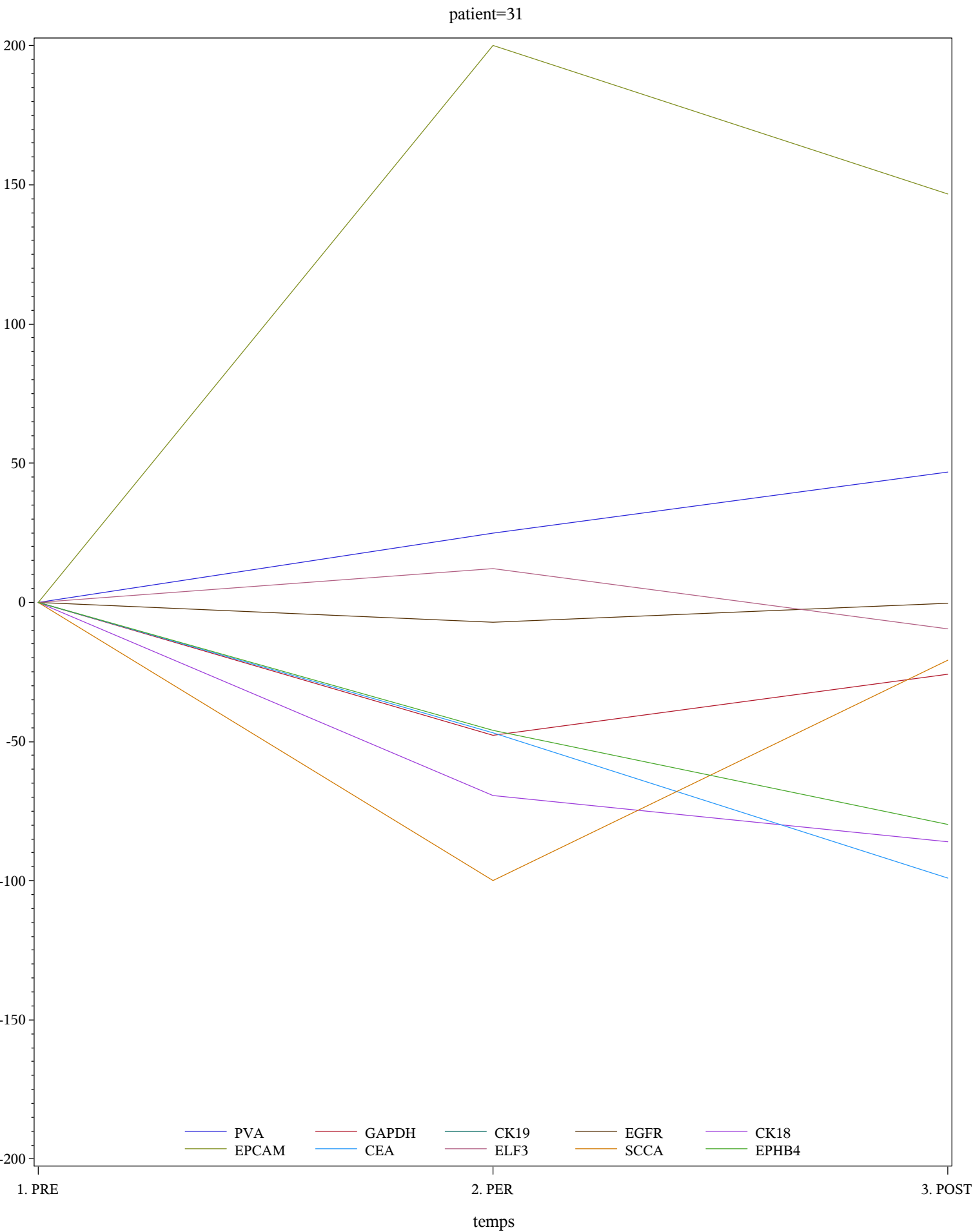

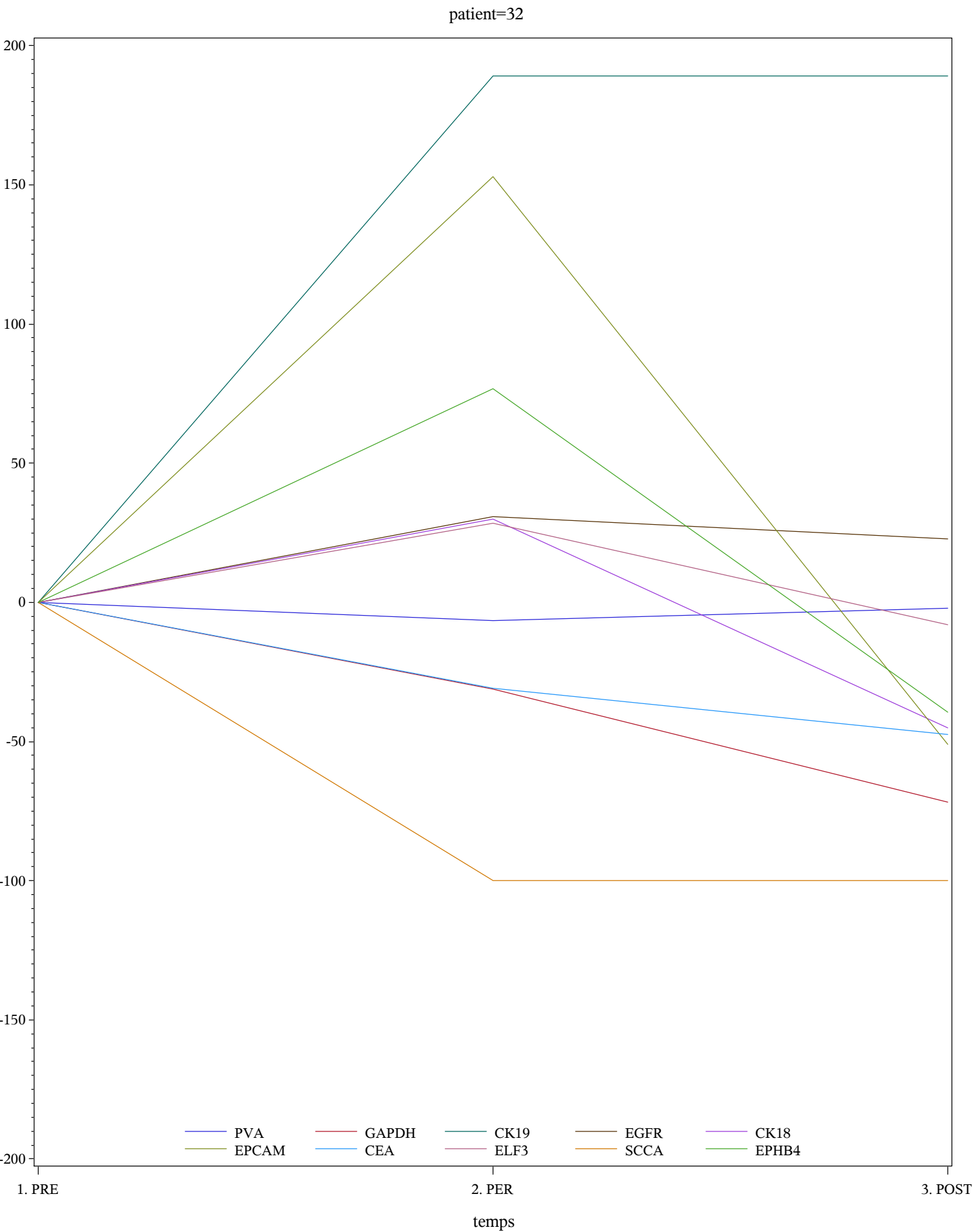

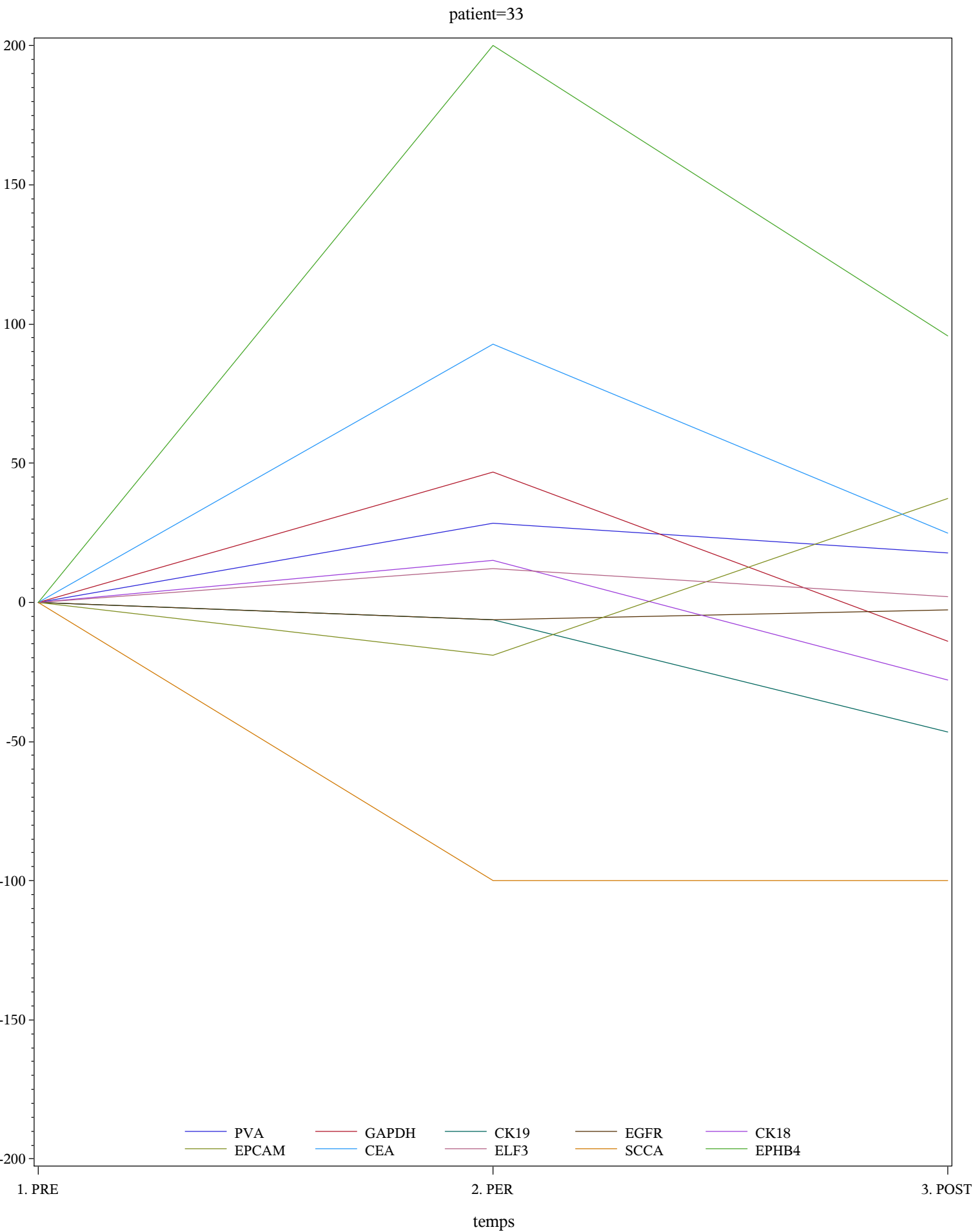

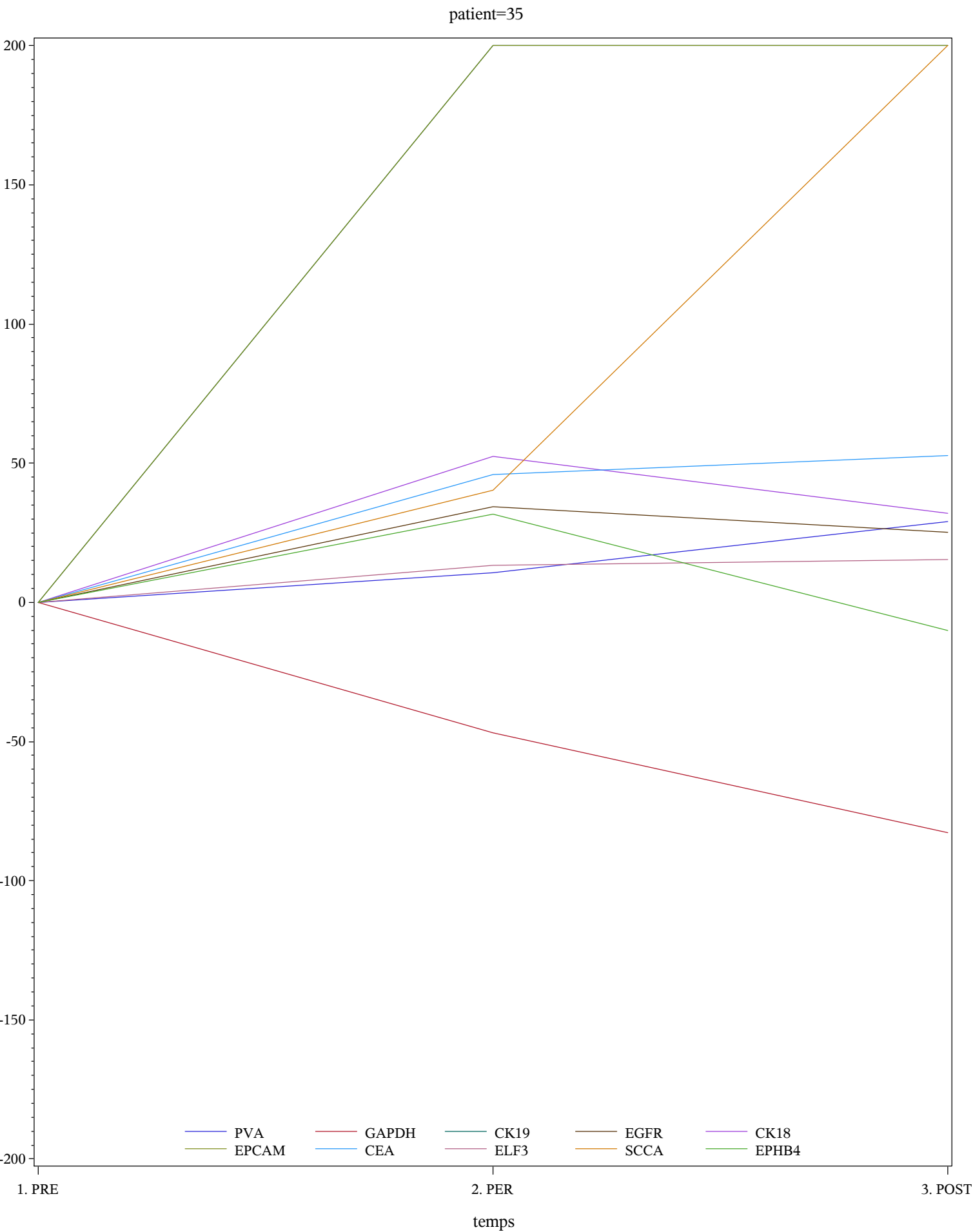

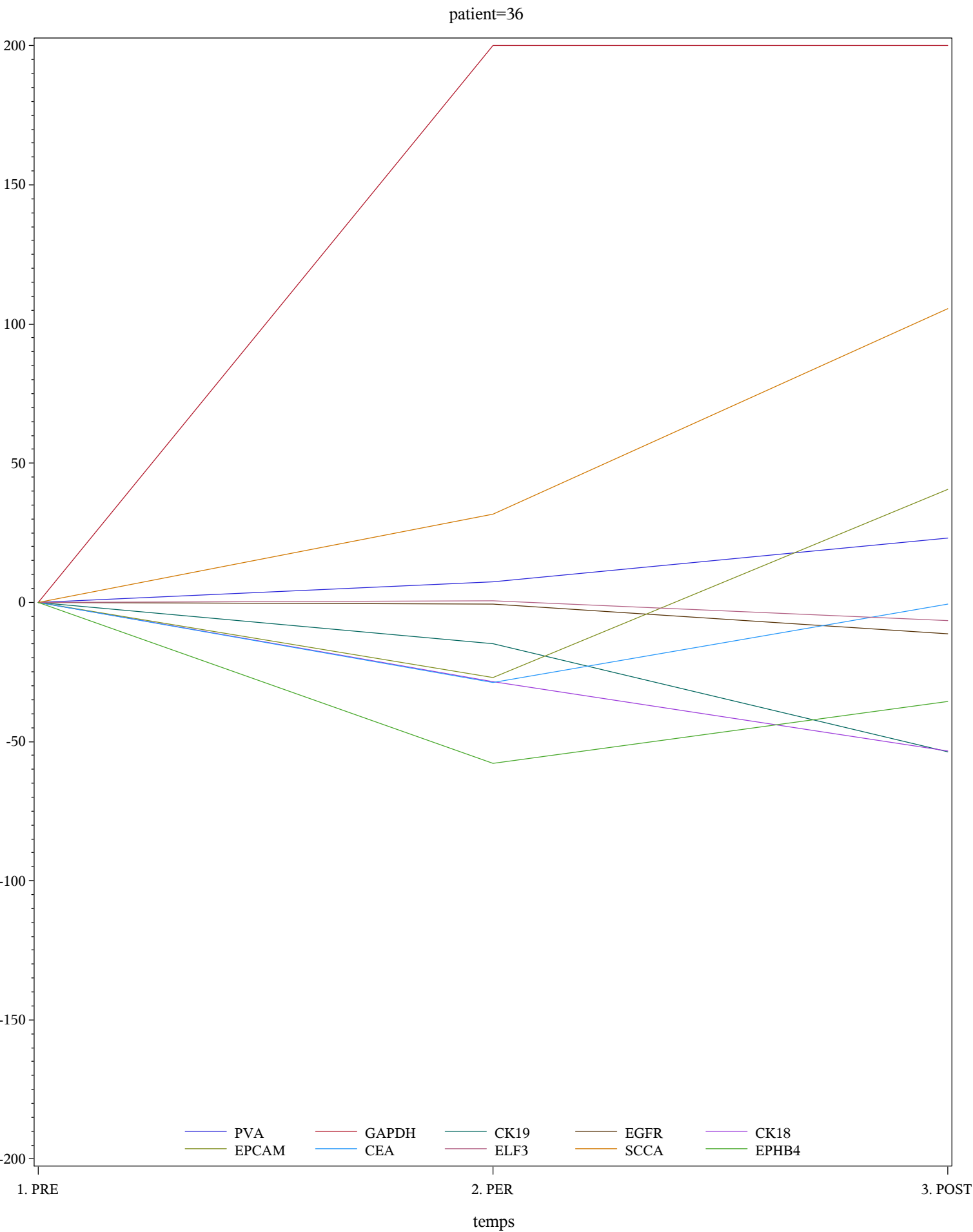

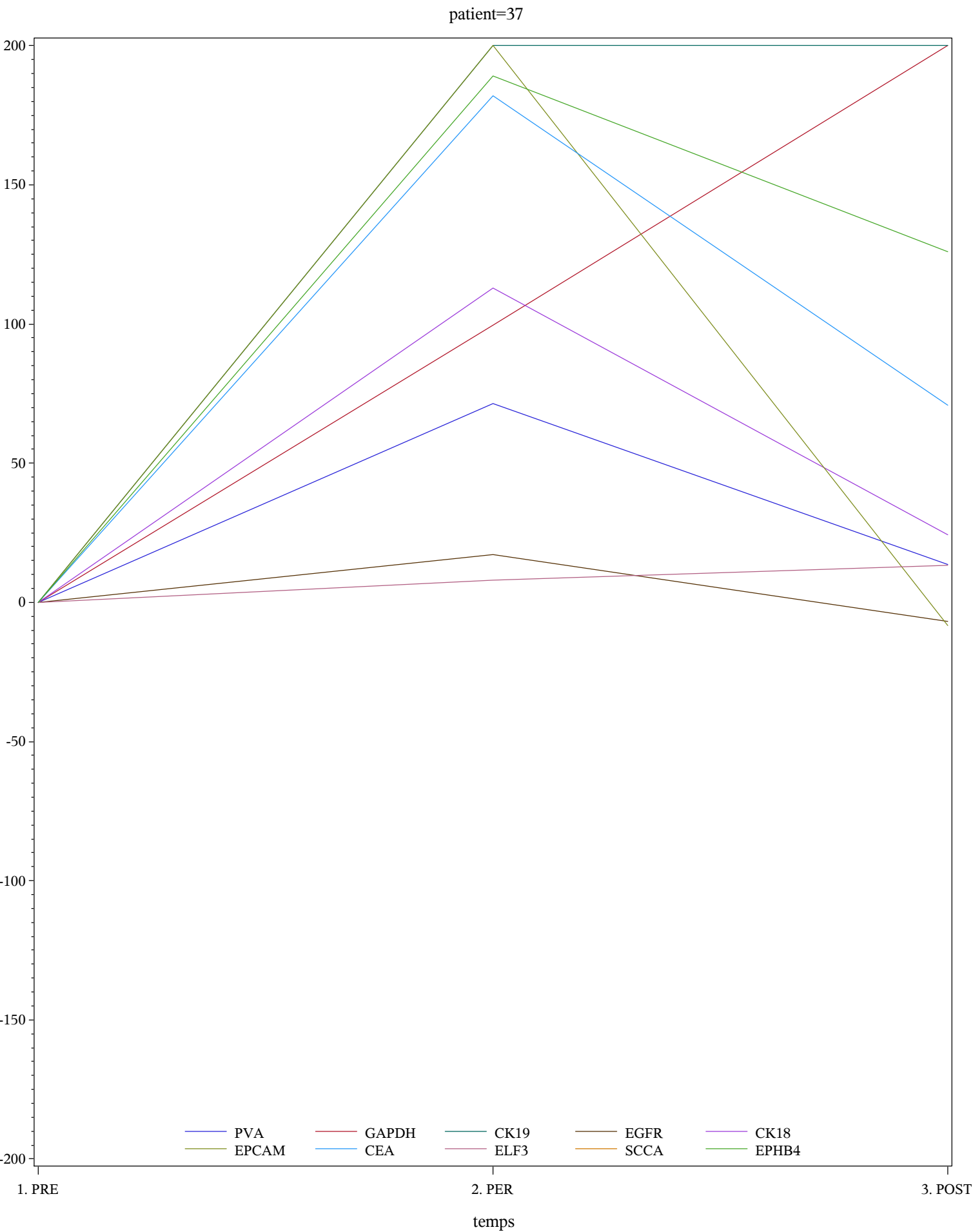

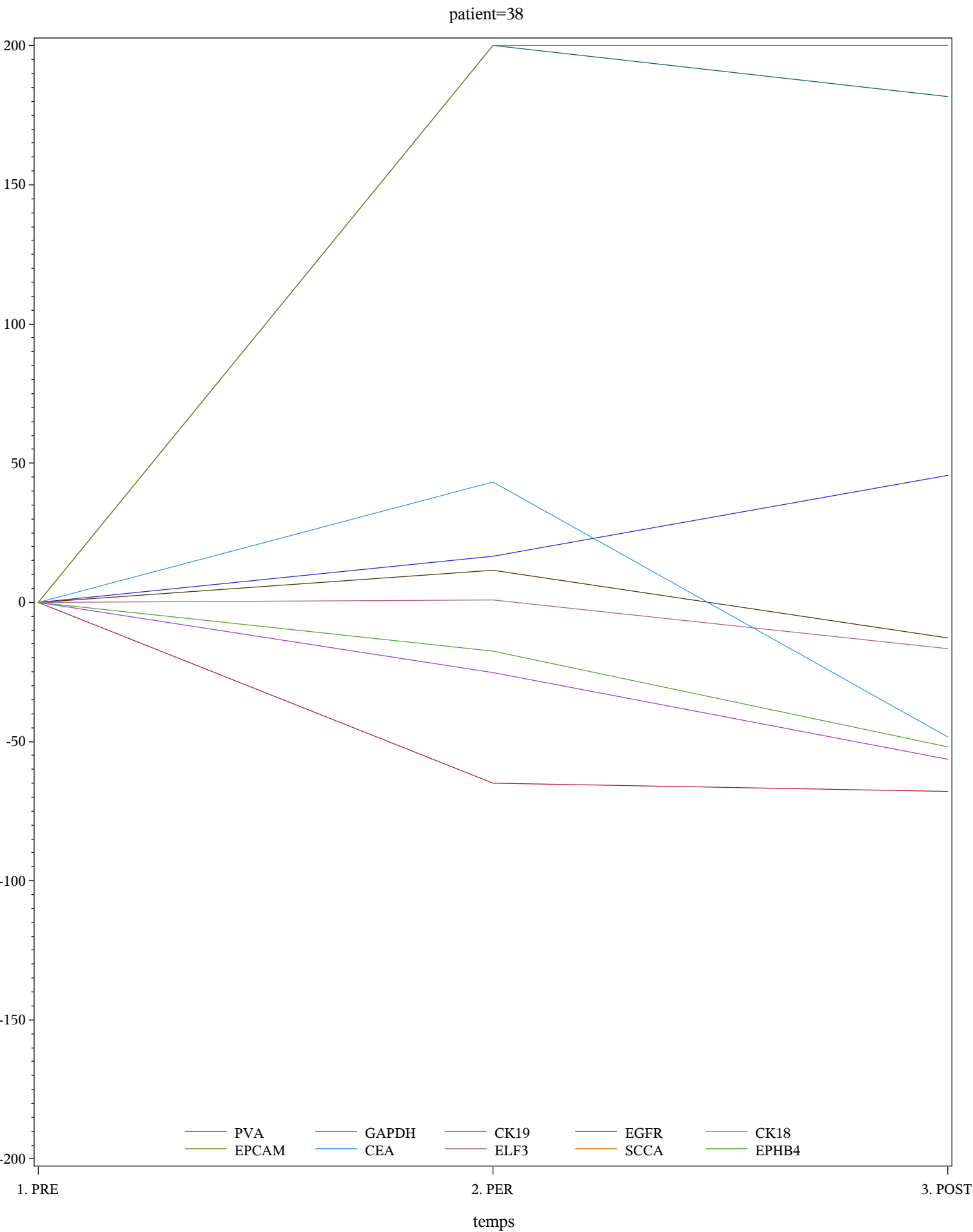

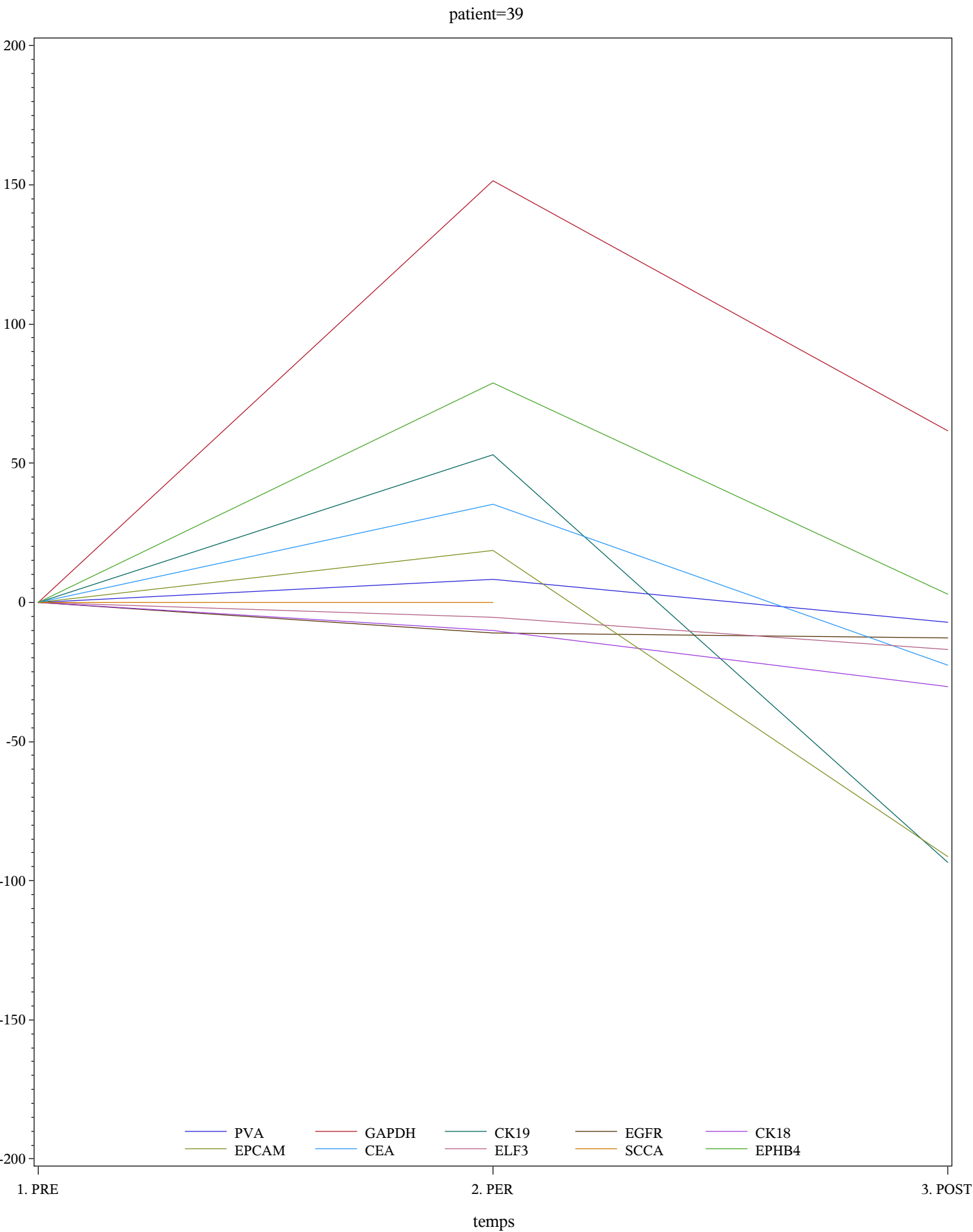

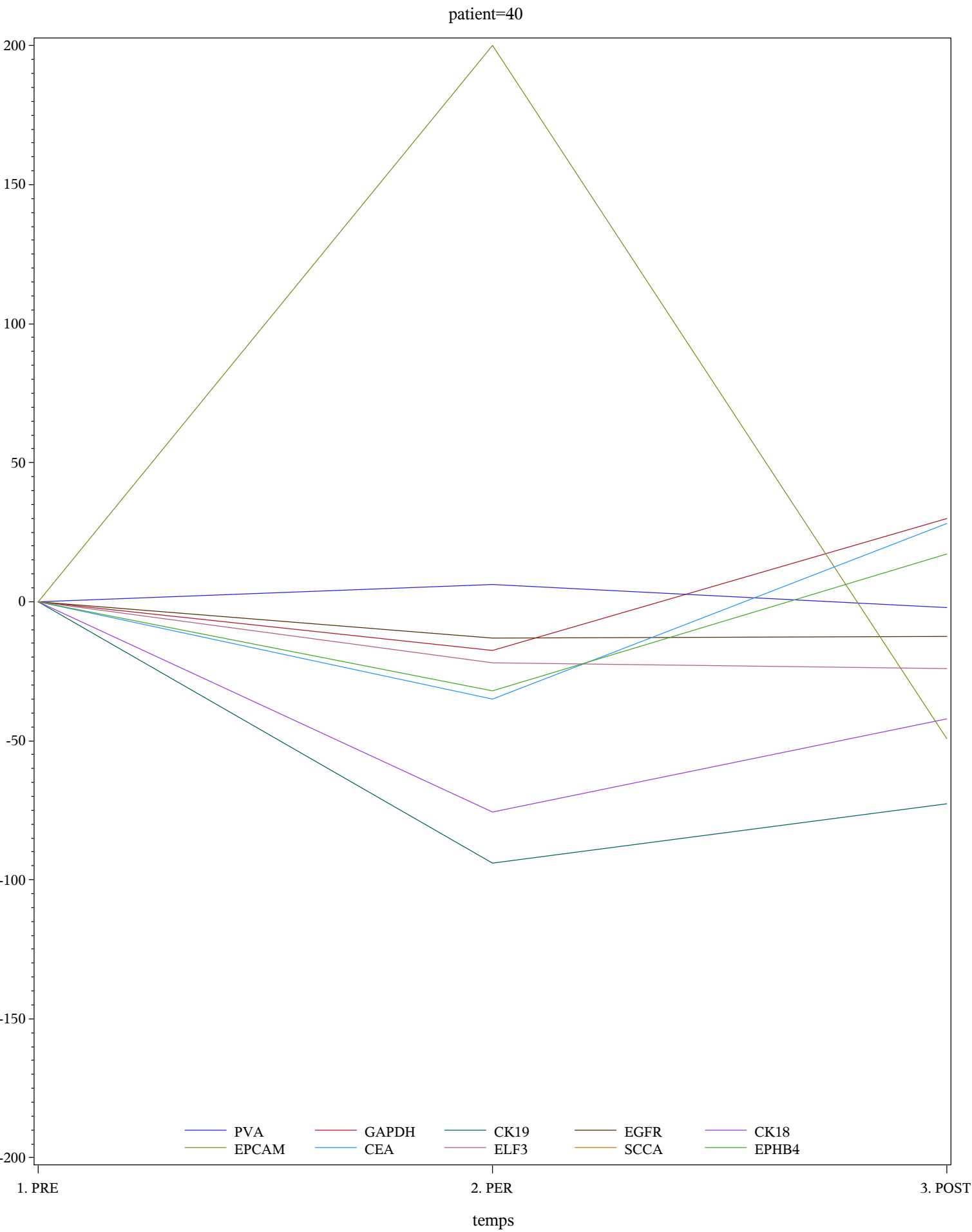

Supplement: S1 File — (PDF) [file pone.0320485.s001.pdf]
