# Supplementary material for: Study of the survival of patients with head and neck cancer in relation to Circulating Tumor Cells (CTCs)
Source: PLoS One. 2025 Apr 1;20(4):e0320485. doi: 10.1371/journal.pone.0320485 (PMC11960953; doi:10.1371/journal.pone.0320485)
Supplement: S2 File — (PDF) [file pone.0320485.s002.pdf]

celltracks analyzer II®

RAPPORT DE RECHERCHE

Établissement : CHU Nancy Brabois
Plate-Forme Nancytomique

Date du rapport : 15/05/2015 14:24

ID échantillon : A-P 001
Volume : 7.5 ml

ID patient :

ID cartouche : 01383844
N° d'analyse : 1

Instruments et opérateurs

CellTracks Analyzer II®

N° de série : CT0811031
Protocole du test : CTC EGFr
ID de l'opérateur d'analyse : labo
Date/heure de l'analyse : 08/04/2015 13:21
ID du premier réviseur : labo
Date/heure de la revue : 08/04/2015 14:05
ID du dernier réviseur : labo
Date/heure de la revue : 08/04/2015 14:20

CellTracks® AutoPrep® System

N° de série : AP0811025
ID opérateur : labo
Date de préparation : 08/04/2015
Heure de préparation : 12:26
Position de l'échantillon : 1
Date du prélèvement :
Date du prélèvement :

Informations sur le lot

Trousse de réactifs

ID de la trousse : CellSearch® CTC
Lot de la trousse : 0074B
Date de péremption : 08/10/2015

Réactif de référence

ID marqueur : EGFr
Lot du marqueur : B775P_
Date de péremption : 17/09/2015

Résultats

Réservé à la recherche. Ne pas utiliser dans le cadre de procédures diagnostiques.

Résultat	Nb de cellules	% de cellules
CTC+:	15	100.00
CTC+/EGFr+:	1	6.67
CTC+/EGFr-:	14	93.33
Non affectés :	798	

Commentaires

CellTracks Analyzer II® Commentaires - 1 EGFr+

Autorisation du rapport : ______________________ Date : ______________

celltracks analyzer II®

RAPPORT DE RECHERCHE

Établissement : CHU Nancy Brabois
Plate-Forme Nancytomique

Date du rapport : 15/05/2015 14:24

ID échantillon : A-P 002
Volume : 7.5 ml

ID patient :

ID cartouche : 01383863
N° d'analyse : 1

Instruments et opérateurs

CellTracks Analyzer II®

N° de série : CT0811031
Protocole du test : CTC EGFr
ID de l'opérateur d'analyse : labo
Date/heure de l'analyse : 08/04/2015 13:35
ID du premier réviseur : labo
Date/heure de la revue : 08/04/2015 14:22
ID du dernier réviseur : labo
Date/heure de la revue : 08/04/2015 14:24

CellTracks® AutoPrep® System

N° de série : AP0811025
ID opérateur : labo
Date de préparation : 08/04/2015
Heure de préparation : 12:39
Position de l'échantillon : 2
Date du prélèvement :
Date du prélèvement :

Informations sur le lot

Trousse de réactifs

ID de la trousse : CellSearch® CTC
Lot de la trousse : 0074B
Date de péremption : 08/10/2015

Réactif de référence

ID marqueur : EGFr
Lot du marqueur : B775P_
Date de péremption : 17/09/2015

Résultats

Réservé à la recherche. Ne pas utiliser dans le cadre de procédures diagnostiques.

Résultat	Nb de cellules	% de cellules
CTC+:	3	100.00
CTC+/EGFr+:	0	0.00
CTC+/EGFr-:	3	100.00
Non affectés :	164	

Commentaires

Autorisation du rapport : ______________________ Date : ______________

celltracks analyzer II®

RAPPORT DE RECHERCHE

Établissement : CHU Nancy Brabois
Plate-Forme Nancytomique

Date du rapport : 15/05/2015 14:23

ID échantillon : A-P 003
Volume : 7.5 ml

ID patient :

ID cartouche : 01376664
N° d'analyse : 1

Instruments et opérateurs

CellTracks Analyzer II®

N° de série : CT0811031
Protocole du test : CTC EGFr
ID de l'opérateur d'analyse : labo
Date/heure de l'analyse : 15/04/2015 14:10
ID du premier réviseur : labo
Date/heure de la revue : 15/04/2015 14:51
ID du dernier réviseur : labo
Date/heure de la revue : 15/04/2015 14:55

CellTracks® AutoPrep® System

N° de série : AP0811025
ID opérateur : labo
Date de préparation : 15/04/2015
Heure de préparation : 13:46
Position de l'échantillon : 1
Date du prélèvement :
Date du prélèvement :

Informations sur le lot

Trousse de réactifs

ID de la trousse : CellSearch® CTC
Lot de la trousse : 0074B
Date de péremption : 08/10/2015

Réactif de référence

ID marqueur : EGFr
Lot du marqueur : B775P_
Date de péremption : 17/09/2015

Résultats

Réservé à la recherche. Ne pas utiliser dans le cadre de procédures diagnostiques.

Résultat	Nb de cellules	% de cellules
CTC+:	3	100.00
CTC+/EGFr+:	2	66.67
CTC+/EGFr-:	1	33.33
Non affectés :	168	

Commentaires

Autorisation du rapport : ______________________ Date : ______________

RAPPORT DE RECHERCHE

Établissement : CHU Nancy Brabois
Plate-Forme Nancytomique

Date du rapport : 15/05/2015 14:25

ID échantillon : C-M 001
Volume : 7.5 ml

ID patient :

ID cartouche : 01358662
N° d'analyse : 1

Instruments et opérateurs

CellTracks Analyzer II®

N° de série : CT0811031
Protocole du test : CTC EGFr
ID de l'opérateur d'analyse : labo
Date/heure de l'analyse : 19/03/2015 14:06
ID du premier réviseur : labo
Date/heure de la revue : 19/03/2015 14:33
ID du dernier réviseur : labo
Date/heure de la revue : 19/03/2015 14:34

CellTracks® AutoPrep® System

N° de série : AP0811025
ID opérateur : labo
Date de préparation : 19/03/2015
Heure de préparation : 13:37
Position de l'échantillon : 1
Date du prélèvement :
Date du prélèvement :

Informations sur le lot

Trousse de réactifs

ID de la trousse : CellSearch® CTC
Lot de la trousse : 0054A
Date de péremption : 02/05/2015

Réactif de référence

ID marqueur : EGFr
Lot du marqueur : B775P_
Date de péremption : 17/09/2015

Résultats

Réservé à la recherche. Ne pas utiliser dans le cadre de procédures diagnostiques.

Résultat	Nb de cellules	% de cellules
CTC+:	2	100.00
CTC+/EGFr+:	0	0.00
CTC+/EGFr-:	2	100.00
Non affectés :	45	

Commentaires

Autorisation du rapport : ____________________ Date : ____________________

celltracks analyzer II®

RAPPORT DE RECHERCHE

Établissement : CHU Nancy Brabois
Plate-Forme Nancytomique

Date du rapport : 15/05/2015 14:25

ID échantillon : C-M 002
Volume : 7.5 ml

ID patient :

ID cartouche : 01358667
N° d'analyse : 1

Instruments et opérateurs

CellTracks Analyzer II®

N° de série : CT0811031
Protocole du test : CTC EGFr
ID de l'opérateur d'analyse : labo
Date/heure de l'analyse : 19/03/2015 14:19
ID du premier réviseur : labo
Date/heure de la revue : 19/03/2015 14:42
ID du dernier réviseur : labo
Date/heure de la revue : 19/03/2015 14:42

CellTracks® AutoPrep® System

N° de série : AP0811025
ID opérateur : labo
Date de préparation : 19/03/2015
Heure de préparation : 13:49
Position de l'échantillon : 2
Date du prélèvement :
Date du prélèvement :

Informations sur le lot

Trousse de réactifs

ID de la trousse : CellSearch® CTC
Lot de la trousse : 0054A
Date de péremption : 02/05/2015

Réactif de référence

ID marqueur : EGFr
Lot du marqueur : B775P_
Date de péremption : 17/09/2015

Résultats

Réservé à la recherche. Ne pas utiliser dans le cadre de procédures diagnostiques.

Résultat	Nb de cellules	% de cellules
CTC+:	0	0.00
CTC+/EGFr+:	0	0.00
CTC+/EGFr-:	0	0.00
Non affectés :	28	

Commentaires

Autorisation du rapport : ____________________ Date : ____________________

RAPPORT DE RECHERCHE

Établissement : CHU Nancy Brabois
Plate-Forme Nancytomique

Date du rapport : 15/05/2015 14:24

ID échantillon : C-M 003
Volume : 7.5 ml
ID patient :
ID cartouche : 01358663
N° d'analyse : 1

Instruments et opérateurs

CellTracks Analyzer II®

N° de série : CT0811031
Protocole du test : CTC EGFr
ID de l'opérateur d'analyse : labo
Date/heure de l'analyse : 27/03/2015 13:45
ID du premier réviseur : labo
Date/heure de la revue : 27/03/2015 14:08
ID du dernier réviseur : labo
Date/heure de la revue : 07/04/2015 13:31

CellTracks® AutoPrep® System

N° de série : AP0811025
ID opérateur : labo
Date de préparation : 27/03/2015
Heure de préparation : 13:20
Position de l'échantillon : 1
Date du prélèvement :
Date du prélèvement :

Informations sur le lot

Trousse de réactifs

ID de la trousse : CellSearch® CTC
Lot de la trousse : 0054A
Date de péremption : 02/05/2015

Réactif de référence

ID marqueur : EGFr
Lot du marqueur : B775P_
Date de péremption : 17/09/2015

Résultats

Réservé à la recherche. Ne pas utiliser dans le cadre de procédures diagnostiques.

Résultat	Nb de cellules	% de cellules
CTC+:	2	100.00
CTC+/EGFr+:	2	100.00
CTC+/EGFr-:	0	0.00
Non affectés :	53	

Commentaires

Autorisation du rapport : ____________________ Date : ____________

celltracks analyzer II®

RAPPORT DE RECHERCHE

Établissement : CHU Nancy Brabois
Plate-Forme Nancytomique

Date du rapport : 15/05/2015 14:23

ID échantillon : D-R 001
Volume : 7.5 ml

ID patient :

ID cartouche : 01376630
N° d'analyse : 1

Instruments et opérateurs

CellTracks Analyzer II®

N° de série : CT0811031
Protocole du test : CTC EGFr
ID de l'opérateur d'analyse : labo
Date/heure de l'analyse : 30/04/2015 13:53
ID du premier réviseur : labo
Date/heure de la revue : 30/04/2015 14:35
ID du dernier réviseur : labo
Date/heure de la revue : 30/04/2015 14:36

CellTracks® AutoPrep® System

N° de série : AP0811025
ID opérateur : labo
Date de préparation : 30/04/2015
Heure de préparation : 12:44
Position de l'échantillon : 1
Date du prélèvement :
Date du prélèvement :

Informations sur le lot

Trousse de réactifs

ID de la trousse : CellSearch® CTC
Lot de la trousse : 0074B
Date de péremption : 08/10/2015

Réactif de référence

ID marqueur : EGFr
Lot du marqueur : B775P_
Date de péremption : 17/09/2015

Résultats

Réservé à la recherche. Ne pas utiliser dans le cadre de procédures diagnostiques.

Résultat	Nb de cellules	% de cellules
CTC+:	1	100.00
CTC+/EGFr+:	1	100.00
CTC+/EGFr-:	0	0.00
Non affectés :	71	

Commentaires

Autorisation du rapport : ______________________ Date : ______________

celltracks analyzer II®

RAPPORT DE RECHERCHE

Établissement : CHU Nancy Brabois
Plate-Forme Nancytomique

Date du rapport : 15/05/2015 14:21

ID échantillon : D-R 002
Volume : 7.5 ml

ID patient :

ID cartouche : 01356713
N° d'analyse : 1

Instruments et opérateurs

CellTracks Analyzer II®

N° de série : CT0811031
Protocole du test : CTC EGFr
ID de l'opérateur d'analyse : labo
Date/heure de l'analyse : 30/04/2015 14:06
ID du premier réviseur : labo
Date/heure de la revue : 30/04/2015 14:36
ID du dernier réviseur : labo
Date/heure de la revue : 30/04/2015 14:37

CellTracks® AutoPrep® System

N° de série : AP0811025
ID opérateur : labo
Date de préparation : 30/04/2015
Heure de préparation : 12:57
Position de l'échantillon : 2
Date du prélèvement :
Date du prélèvement :

Informations sur le lot

Trousse de réactifs

ID de la trousse : CellSearch® CTC
Lot de la trousse : 0074B
Date de péremption : 08/10/2015

Réactif de référence

ID marqueur : EGFr
Lot du marqueur : B775P_
Date de péremption : 17/09/2015

Résultats

Réservé à la recherche. Ne pas utiliser dans le cadre de procédures diagnostiques.

Résultat	Nb de cellules	% de cellules
CTC+:	1	100.00
CTC+/EGFr+:	0	0.00
CTC+/EGFr-:	1	100.00
Non affectés :	46	

Commentaires

Autorisation du rapport : ____________________ Date : ____________________

celltracks analyzer II®

RAPPORT DE RECHERCHE

Établissement : CHU Nancy Brabois
Plate-Forme Nancytomique

Date du rapport : 15/05/2015 14:03

ID échantillon : D-R 003
Volume : 7.5 ml

ID patient :

ID cartouche : 01356710
N° d'analyse : 1

Instruments et opérateurs

CellTracks Analyzer II®

N° de série : CT0811031
Protocole du test : CTC EGFr
ID de l'opérateur d'analyse : labo
Date/heure de l'analyse : 07/05/2015 15:12
ID du premier réviseur : labo
Date/heure de la revue : 07/05/2015 15:54
ID du dernier réviseur : labo
Date/heure de la revue : 07/05/2015 15:54

CellTracks® AutoPrep® System

N° de série : AP0811025
ID opérateur : labo
Date de préparation : 07/05/2015
Heure de préparation : 13:53
Position de l'échantillon : 3
Date du prélèvement :
Date du prélèvement :

Informations sur le lot

Trousse de réactifs

ID de la trousse : CellSearch® CTC
Lot de la trousse : 0074B
Date de péremption : 08/10/2015

Réactif de référence

ID marqueur : EGFr
Lot du marqueur : B775P_
Date de péremption : 17/09/2015

Résultats

Réservé à la recherche. Ne pas utiliser dans le cadre de procédures diagnostiques.

Résultat	Nb de cellules	% de cellules
CTC+:	0	0.00
CTC+/EGFr+:	0	0.00
CTC+/EGFr-:	0	0.00
Non affectés :	65	

Commentaires

Autorisation du rapport : ______________________ Date : ______________

celltracks analyzer II®

RAPPORT DE RECHERCHE

Établissement : CHU Nancy Brabois
Plate-Forme Nancytomique

Date du rapport : 15/05/2015 14:25

ID échantillon : F-M 002
Volume : 7.5 ml

ID patient :

ID cartouche : 01319422
N° d'analyse : 1

Instruments et opérateurs

CellTracks Analyzer II®

N° de série : CT0811031
Protocole du test : CTC EGFr
ID de l'opérateur d'analyse : labo
Date/heure de l'analyse : 01/01/2002 00:27
ID du premier réviseur : labo
Date/heure de la revue : 09/03/2015 15:07
ID du dernier réviseur : labo
Date/heure de la revue : 09/03/2015 15:10

CellTracks® AutoPrep® System

N° de série : AP0811025
ID opérateur : labo
Date de préparation : 09/03/2015
Heure de préparation : 13:39
Position de l'échantillon : 1
Date du prélèvement :
Date du prélèvement :

Informations sur le lot

Trousse de réactifs

ID de la trousse : CellSearch® CTC
Lot de la trousse : 0054A
Date de péremption : 02/05/2015

Réactif de référence

ID marqueur : EGFr
Lot du marqueur : B775P_
Date de péremption : 17/09/2015

Résultats

Réservé à la recherche. Ne pas utiliser dans le cadre de procédures diagnostiques.

Résultat	Nb de cellules	% de cellules
CTC+:	1	100.00
CTC+/EGFr+:	0	0.00
CTC+/EGFr-:	1	100.00
Non affectés :	46	

Commentaires

Autorisation du rapport : ______________________ Date : ______________

RAPPORT DE RECHERCHE

celltracks analyzer II®

Établissement : CHU Nancy Brabois
Plate-Forme Nancytomique

Date du rapport : 15/05/2015 14:25

ID échantillon : F-M 003
Volume : 7.5 ml

ID patient :

ID cartouche : 01358666
N° d'analyse : 1

Instruments et opérateurs

CellTracks Analyzer II®

N° de série : CT0811031
Protocole du test : CTC EGFr
ID de l'opérateur d'analyse : labo
Date/heure de l'analyse : 16/03/2015 14:46
ID du premier réviseur : labo
Date/heure de la revue : 16/03/2015 15:12
ID du dernier réviseur : labo
Date/heure de la revue : 16/03/2015 15:12

CellTracks® AutoPrep® System

N° de série : AP0811025
ID opérateur : labo
Date de préparation : 16/03/2015
Heure de préparation : 14:10
Position de l'échantillon : 1
Date du prélèvement :
Date du prélèvement :

Informations sur le lot

Trousse de réactifs

ID de la trousse : CellSearch® CTC
Lot de la trousse : 0054A
Date de péremption : 02/05/2015

Réactif de référence

ID marqueur : EGFr
Lot du marqueur : B775P_
Date de péremption : 17/09/2015

Résultats

Réservé à la recherche. Ne pas utiliser dans le cadre de procédures diagnostiques.

Résultat	Nb de cellules	% de cellules
CTC+:	0	0.00
CTC+/EGFr+:	0	0.00
CTC+/EGFr-:	0	0.00
Non affectés :	106	

Commentaires

Autorisation du rapport : ______________________ Date : ______________

celltracks analyzer II®

RAPPORT DE RECHERCHE

Établissement : CHU Nancy Brabois
Plate-Forme Nancytomique

Date du rapport : 15/05/2015 14:03

ID échantillon : G-D 001
Volume : 7.5 ml

ID patient :

ID cartouche : 01356711
N° d'analyse : 1

Instruments et opérateurs

CellTracks Analyzer II®

N° de série : CT0811031
Protocole du test : CTC EGFr
ID de l'opérateur d'analyse : labo
Date/heure de l'analyse : 07/05/2015 14:45
ID du premier réviseur : labo
Date/heure de la revue : 07/05/2015 15:48
ID du dernier réviseur : labo
Date/heure de la revue : 07/05/2015 15:52

CellTracks® AutoPrep® System

N° de série : AP0811025
ID opérateur : labo
Date de préparation : 07/05/2015
Heure de préparation : 13:27
Position de l'échantillon : 1
Date du prélèvement :
Date du prélèvement :

Informations sur le lot

Trousse de réactifs

ID de la trousse : CellSearch® CTC
Lot de la trousse : 0074B
Date de péremption : 08/10/2015

Réactif de référence

ID marqueur : EGFr
Lot du marqueur : B775P_
Date de péremption : 17/09/2015

Résultats

Réservé à la recherche. Ne pas utiliser dans le cadre de procédures diagnostiques.

Résultat	Nb de cellules	% de cellules
CTC+:	5	100.00
CTC+/EGFr+:	5	100.00
CTC+/EGFr-:	0	0.00
Non affectés :	198	

Commentaires

Autorisation du rapport : ______________________ Date : ______________

RAPPORT DE RECHERCHE

celltracks analyzer II®

Établissement : CHU Nancy Brabois
Plate-Forme Nancytomique

Date du rapport : 15/05/2015 14:03

ID échantillon : G-D 002
Volume : 7.5 ml

ID patient :

ID cartouche : 01356708
N° d'analyse : 1

Instruments et opérateurs

CellTracks Analyzer II®

N° de série : CT0811031
Protocole du test : CTC EGFr
ID de l'opérateur d'analyse : labo
Date/heure de l'analyse : 07/05/2015 14:59
ID du premier réviseur : labo
Date/heure de la revue : 07/05/2015 15:52
ID du dernier réviseur : labo
Date/heure de la revue : 07/05/2015 15:52

CellTracks® AutoPrep® System

N° de série : AP0811025
ID opérateur : labo
Date de préparation : 07/05/2015
Heure de préparation : 13:40
Position de l'échantillon : 2
Date du prélèvement :
Date du prélèvement :

Informations sur le lot

Trousse de réactifs

ID de la trousse : CellSearch® CTC
Lot de la trousse : 0074B
Date de péremption : 08/10/2015

Réactif de référence

ID marqueur : EGFr
Lot du marqueur : B775P_
Date de péremption : 17/09/2015

Résultats

Réservé à la recherche. Ne pas utiliser dans le cadre de procédures diagnostiques.

Résultat	Nb de cellules	% de cellules
CTC+:	0	0.00
CTC+/EGFr+:	0	0.00
CTC+/EGFr-:	0	0.00
Non affectés :	30	

Commentaires

Autorisation du rapport : ______________________ Date : ______________

celltracks analyzer II®

RAPPORT DE RECHERCHE

Établissement : CHU Nancy Brabois
Plate-Forme Nancytomique

Date du rapport : 15/05/2015 14:03

ID échantillon : G-D 003
Volume : 7.5 ml

ID patient :

ID cartouche : 01356716
N° d'analyse : 1

Instruments et opérateurs

CellTracks Analyzer II®

N° de série : CT0811031
Protocole du test : CTC EGFr
ID de l'opérateur d'analyse : labo
Date/heure de l'analyse : 13/05/2015 13:39
ID du premier réviseur : labo
Date/heure de la revue : 13/05/2015 14:01
ID du dernier réviseur : labo
Date/heure de la revue : 13/05/2015 14:02

CellTracks® AutoPrep® System

N° de série : AP0811025
ID opérateur : labo
Date de préparation : 13/05/2015
Heure de préparation : 12:25
Position de l'échantillon : 1
Date du prélèvement :
Date du prélèvement :

Informations sur le lot

Trousse de réactifs

ID de la trousse : CellSearch® CTC
Lot de la trousse : 0074B
Date de péremption : 08/10/2015

Réactif de référence

ID marqueur : EGFr
Lot du marqueur : B775P_
Date de péremption : 17/09/2015

Résultats

Réservé à la recherche. Ne pas utiliser dans le cadre de procédures diagnostiques.

Résultat	Nb de cellules	% de cellules
CTC+:	1	100.00
CTC+/EGFr+:	1	100.00
CTC+/EGFr-:	0	0.00
Non affectés :	24	

Commentaires

Autorisation du rapport : ______________________ Date : ______________

celltracks analyzer II®

RAPPORT DE RECHERCHE

Établissement : CHU Nancy Brabois
Plate-Forme Nancytomique

Date du rapport : 15/05/2015 14:24

ID échantillon : H-J 001
Volume : 7.5 ml

ID patient :

ID cartouche : 01376663
N° d'analyse : 1

Instruments et opérateurs

CellTracks Analyzer II®

N° de série : CT0811031
Protocole du test : CTC EGFr
ID de l'opérateur d'analyse : labo
Date/heure de l'analyse : 13/04/2015 13:57
ID du premier réviseur : labo
Date/heure de la revue : 13/04/2015 15:03
ID du dernier réviseur : labo
Date/heure de la revue : 13/04/2015 15:03

CellTracks® AutoPrep® System

N° de série : AP0811025
ID opérateur : labo
Date de préparation : 13/04/2015
Heure de préparation : 13:34
Position de l'échantillon : 2
Date du prélèvement :
Date du prélèvement :

Informations sur le lot

Trousse de réactifs

ID de la trousse : CellSearch® CTC
Lot de la trousse : 0074B
Date de péremption : 08/10/2015

Réactif de référence

ID marqueur : EGFr
Lot du marqueur : B775P_
Date de péremption : 17/09/2015

Résultats

Réservé à la recherche. Ne pas utiliser dans le cadre de procédures diagnostiques.

Résultat	Nb de cellules	% de cellules
CTC+:	0	0.00
CTC+/EGFr+:	0	0.00
CTC+/EGFr-:	0	0.00
Non affectés :	38	

Commentaires

Autorisation du rapport : ______________________ Date : ______________

celltracks analyzer II®

RAPPORT DE RECHERCHE

Établissement : CHU Nancy Brabois
Plate-Forme Nancytomique

Date du rapport : 15/05/2015 14:23

ID échantillon : H-J 002
Volume : 7.5 ml

ID patient :

ID cartouche : 01383861
N° d'analyse : 1

Instruments et opérateurs

CellTracks Analyzer II®

N° de série : CT0811031
Protocole du test : CTC EGFr
ID de l'opérateur d'analyse : labo
Date/heure de l'analyse : 13/04/2015 14:12
ID du premier réviseur : labo
Date/heure de la revue : 13/04/2015 15:03
ID du dernier réviseur : labo
Date/heure de la revue : 13/04/2015 15:04

CellTracks® AutoPrep® System

N° de série : AP0811025
ID opérateur : labo
Date de préparation : 13/04/2015
Heure de préparation : 13:47
Position de l'échantillon : 3
Date du prélèvement :
Date du prélèvement :

Informations sur le lot

Trousse de réactifs

ID de la trousse : CellSearch® CTC
Lot de la trousse : 0074B
Date de péremption : 08/10/2015

Réactif de référence

ID marqueur : EGFr
Lot du marqueur : B775P_
Date de péremption : 17/09/2015

Résultats

Réservé à la recherche. Ne pas utiliser dans le cadre de procédures diagnostiques.

Résultat	Nb de cellules	% de cellules
CTC+:	2	100.00
CTC+/EGFr+:	2	100.00
CTC+/EGFr-:	0	0.00
Non affectés :	52	

Commentaires

Autorisation du rapport : ______________________ Date : ______________

RAPPORT DE RECHERCHE

Établissement : CHU Nancy Brabois
Plate-Forme Nancytomique

Date du rapport : 15/05/2015 14:23

ID échantillon : H-J 003
Volume : 7.5 ml

ID patient :

ID cartouche : 01376637
N° d'analyse : 1

Instruments et opérateurs

CellTracks Analyzer II®

N° de série : CT0811031
Protocole du test : CTC EGFr
ID de l'opérateur d'analyse : labo
Date/heure de l'analyse : 17/04/2015 16:29
ID du premier réviseur : labo
Date/heure de la revue : 17/04/2015 17:07
ID du dernier réviseur : labo
Date/heure de la revue : 17/04/2015 17:07

CellTracks® AutoPrep® System

N° de série : AP0811025
ID opérateur : labo
Date de préparation : 17/04/2015
Heure de préparation : 14:02
Position de l'échantillon : 1
Date du prélèvement :
Date du prélèvement :

Informations sur le lot

Trousse de réactifs

ID de la trousse : CellSearch® CTC
Lot de la trousse : 0074B
Date de péremption : 08/10/2015

Réactif de référence

ID marqueur : EGFr
Lot du marqueur : B775P_
Date de péremption : 17/09/2015

Résultats

Réservé à la recherche. Ne pas utiliser dans le cadre de procédures diagnostiques.

Résultat	Nb de cellules	% de cellules
CTC+:	0	0.00
CTC+/EGFr+:	0	0.00
CTC+/EGFr-:	0	0.00
Non affectés :	32	

Commentaires

Autorisation du rapport : ______________________ Date : ______________

RAPPORT DE RECHERCHE

**Établissement :**	CHU Nancy Brabois<br>Plate-Forme Nancytomique	**Date du rapport :**	15/05/2015 14:24

**ID échantillon :**	N-H 001	**ID patient :**		**ID cartouche :**	01358664
**Volume :**	7.5 ml			**N° d'analyse :**	1

Instruments et opérateurs

CellTracks Analyzer II®

N° de série : CT0811031
Protocole du test : CTC EGFr
ID de l'opérateur d'analyse : labo
Date/heure de l'analyse : 07/04/2015 12:53
ID du premier réviseur : labo
Date/heure de la revue : 07/04/2015 13:20
ID du dernier réviseur : labo
Date/heure de la revue : 07/04/2015 13:31

CellTracks® AutoPrep® System

N° de série : AP0811025
ID opérateur : labo
Date de préparation : 07/04/2015
Heure de préparation : 12:16
Position de l'échantillon : 1
Date du prélèvement :
Date du prélèvement :

Informations sur le lot

Trousse de réactifs

ID de la trousse : CellSearch® CTC
Lot de la trousse : 0054A
Date de péremption : 02/05/2015

Réactif de référence

ID marqueur : EGFr
Lot du marqueur : B775P_
Date de péremption : 17/09/2015

Résultats

Réservé à la recherche. Ne pas utiliser dans le cadre de procédures diagnostiques.

Résultat	Nb de cellules	% de cellules
CTC+:	2	100.00
CTC+/EGFr+:	2	100.00
CTC+/EGFr-:	0	0.00
Non affectés :	65	

Commentaires

Autorisation du rapport : ______________________ Date : ______________

RAPPORT DE RECHERCHE

Établissement : CHU Nancy Brabois
Plate-Forme Nancytomique

Date du rapport : 15/05/2015 14:24

ID échantillon : N-H 002
Volume : 7.5 ml

ID patient :

ID cartouche : 01383854
N° d'analyse : 1

Instruments et opérateurs

CellTracks Analyzer II®

N° de série : CT0811031
Protocole du test : CTC EGFr
ID de l'opérateur d'analyse : labo
Date/heure de l'analyse : 07/04/2015 13:07
ID du premier réviseur : labo
Date/heure de la revue : 07/04/2015 13:29
ID du dernier réviseur : labo
Date/heure de la revue : 07/04/2015 13:30

CellTracks® AutoPrep® System

N° de série : AP0811025
ID opérateur : labo
Date de préparation : 07/04/2015
Heure de préparation : 12:28
Position de l'échantillon : 2
Date du prélèvement :
Date du prélèvement :

Informations sur le lot

Trousse de réactifs

ID de la trousse : CellSearch® CTC
Lot de la trousse : 0054A
Date de péremption : 02/05/2015

Réactif de référence

ID marqueur : EGFr
Lot du marqueur : B775P_
Date de péremption : 17/09/2015

Résultats

Réservé à la recherche. Ne pas utiliser dans le cadre de procédures diagnostiques.

Résultat	Nb de cellules	% de cellules
CTC+:	1	100.00
CTC+/EGFr+:	1	100.00
CTC+/EGFr-:	0	0.00
Non affectés :	51	

Commentaires

Autorisation du rapport : ______________________ Date : ______________

celltracks analyzer II®

RAPPORT DE RECHERCHE

Établissement : CHU Nancy Brabois
Plate-Forme Nancytomique

Date du rapport : 15/05/2015 14:23

ID échantillon : N-H 003
Volume : 7.5 ml

ID patient :

ID cartouche : 01383858
N° d'analyse : 1

Instruments et opérateurs

CellTracks Analyzer II®

N° de série : CT0811031
Protocole du test : CTC EGFr
ID de l'opérateur d'analyse : labo
Date/heure de l'analyse : 13/04/2015 14:31
ID du premier réviseur : labo
Date/heure de la revue : 13/04/2015 15:02
ID du dernier réviseur : labo
Date/heure de la revue : 13/04/2015 15:02

CellTracks® AutoPrep® System

N° de série : AP0811025
ID opérateur : labo
Date de préparation : 13/04/2015
Heure de préparation : 13:22
Position de l'échantillon : 1
Date du prélèvement :
Date du prélèvement :

Informations sur le lot

Trousse de réactifs

ID de la trousse : CellSearch® CTC
Lot de la trousse : 0074B
Date de péremption : 08/10/2015

Réactif de référence

ID marqueur : EGFr
Lot du marqueur : B775P_
Date de péremption : 17/09/2015

Résultats

Réservé à la recherche. Ne pas utiliser dans le cadre de procédures diagnostiques.

Résultat	Nb de cellules	% de cellules
CTC+:	0	0.00
CTC+/EGFr+:	0	0.00
CTC+/EGFr-:	0	0.00
Non affectés :	43	

Commentaires

Autorisation du rapport : ____________________ Date : ____________
