# Supplementary material for: Study of the survival of patients with head and neck cancer in relation to Circulating Tumor Cells (CTCs)
Source: PLoS One. 2025 Apr 1;20(4):e0320485. doi: 10.1371/journal.pone.0320485 (PMC11960953; doi:10.1371/journal.pone.0320485)
Supplement: S3 File — (PDF) [file pone.0320485.s003.pdf]

No.	Preoperative		Operative		Postoperative	
	CTC	CTC+EGFr+	CTC	CTC+EGFr+	CTC	CTC+EGFr+
7	1	NA	3	NA	2	NA
9	0	NA	4	0	1	1
10	1	1	1	1	0	0
11	0	0	2	0	0	0
12	4	4	1	1	2	1
13	3	3	0	0	2	1
14	0	0	3	0	NS	NS
15	16	13	2	0	2	0
16	2	1	5	0	2	0
17	4	NA	1	NA	1	0
18	3	1	0	0	9	9
19	4	1	2	1	5	1
20	3	3	9	9	0	0
21	0	0	0	0	0	0
22	0	0	0	0	5	1
23	3	3	15	15	5	2
24	1	1	1	1	2	1
25	3	3	2	1	1	1
26	1	0	1	1	0	0
27	0	0	2	2	0	0
28	0	0	0	0	0	0
29	1	1	0	0	1	1
30	1	0	0	0	0	0
31	2	1	0	0	1	0
32	NS	NS	NS	NS	NS	NS
33	2	2	0	0	1	1
34	2	2	1	1	0	0
35	NC	NC	1	1	0	0
36	15	1	3	0	3	2
37	0	0	2	2	0	0
38	>300	0	>200	0	>1400	0
39	1	1	1	0	0	0
40	5	5	0	0	1	1

Legend:
NA: non reagent available
NS: no sample
NC: nonconformity
